# Bioactive potential of natural biomaterials: identification, retention and assessment of biological properties

**DOI:** 10.1038/s41392-021-00512-8

**Published:** 2021-03-19

**Authors:** Kieran Joyce, Georgina Targa Fabra, Yagmur Bozkurt, Abhay Pandit

**Affiliations:** 1 School of Medicine, National University of Ireland, Galway, Ireland; 2 CÚRAM, SFI Research Centre for Medical Devices, National University of Ireland, Galway, Ireland

**Keywords:** Biomaterials, Biotechnology

## Abstract

Biomaterials have had an increasingly important role in recent decades, in biomedical device design and the development of tissue engineering solutions for cell delivery, drug delivery, device integration, tissue replacement, and more. There is an increasing trend in tissue engineering to use natural substrates, such as macromolecules native to plants and animals to improve the biocompatibility and biodegradability of delivered materials. At the same time, these materials have favourable mechanical properties and often considered to be biologically inert. More importantly, these macromolecules possess innate functions and properties due to their unique chemical composition and structure, which increase their bioactivity and therapeutic potential in a wide range of applications. While much focus has been on integrating these materials into these devices via a spectrum of cross-linking mechanisms, little attention is drawn to residual bioactivity that is often hampered during isolation, purification, and production processes. Herein, we discuss methods of initial material characterisation to determine innate bioactivity, means of material processing including cross-linking, decellularisation, and purification techniques and finally, a biological assessment of retained bioactivity of a final product. This review aims to address considerations for biomaterials design from natural polymers, through the optimisation and preservation of bioactive components that maximise the inherent bioactive potency of the substrate to promote tissue regeneration.

## Introduction

The design and functionalisation of implantable biomaterials have seen significant progress in the last few decades, with an increasing number of implantable biomaterials on the market every year.^1,2^ Biomaterials are widely used in tissue engineering solutions in combination with cells, synthetic materials, and therapeutic molecules to produce advanced therapeutic medicinal products.^3,4^ A three-dimensional polymeric scaffold often provides a support structure for the delivery of cells and biologically actives components. There is an increasing trend in tissue engineering to use naturally occurring macromolecules as a starting material due to their advantageous properties, since such materials are well tolerated, promoting cellular adhesion, and subsequent tissue formation to facilitate body integration while their biodegradability allows for tissue remodelling.^5^ The architecture of an exogenous construct should resemble the interconnecting network of the native tissue for increased cell infiltration, nutrient diffusion, and metabolite elimination.^6^ The mechanical properties of these natural polymers can be modulated to resist compressive forces, match tissue properties, and exert mechanical stimulus on cells.^7^ Innate material biological activity, cell-recognition and subsequent signalling cascades induced by natural polymers are not often highlighted in the scaffold design process, and not well characterised in final scaffold properties and function.^8^

Natural polymers include nucleic acids, polysaccharides, proteins, lipids and complex macromolecules such as proteoglycans. Biological activity may be imparted upon a material through the use of natural polymers derived from non-mammalian and mammalian sources. Cellulose is the most common non-mammalian biopolymer on earth^9^. At the same time, collagens are the most abundant mammalian polymers and the main component of the extracellular matrix (ECM) in most soft and hard mammalian tissues accounting for approximately 30% of all protein mass in the body.^10^ Twenty-eight types of collagens have been identified throughout various tissues where, for example, Collagen type I is most abundant in hard connective tissues like bone and tendon, collagen type II is more abundantly present in articular surfaces, and collagen IV is primarily found in the basal lamina of the basement membrane.^11^ The highly organised architecture of proteins, proteoglycans and polysaccharides in the ECM not only provides mechanical support to tissues, but plays a crucial role in regulating tissue function, cell differentiation, and maintenance of phenotype.^4^

These natural polymers can form non-cytotoxic hydrogels and scaffolds either through self-assembly or using cross-linking techniques to recapitulate natural tissue properties. Fibrous proteins contain highly repetitive amino acid sequences responsible for their unique mechanical properties.^12^ Sequence repetition results in the formation of homogenous-recurring secondary structures (beta-sheets, fibrils, and coils) promoting self-polymerisation and self-assembly into complex, well-organised architectures.^13^ Non-self-assembling polymers can be cross-linked using chemical or physical methods to form stable hydrogels with retained polymeric properties.

This review aims to capture the biological properties of natural macromolecules and ECM components used in tissue engineering to inform biomaterial design for optimal biological performance. We discuss the range of naturally occurring properties in these materials, their initial characterisation, methods of cross-linking and material processing for tuning final biological properties, assessed by biological assays. The design of next-generation implantable materials will require detailed characterisation of modifications such as phosphorylation, sulphation, glycosylation, and other post-translational modifications (PTMs) modulate the structure and cell–matrix interactions in implant-tissue responses. These PTMs enhance receptor and lectin recognition of peptide sequences of protein-based molecules and carbohydrate motifs of polysaccharides on the cell surface, which induce intracellular signalling. These recognition processes are also influenced by ligand availability, which may be masked by the structural organisation of secondary and tertiary structures. A larger structural organisation like molecular weight,^14,15^ fibril formation,^16^ folding and cross-linking,^17^ are crucial for cell response to the material. We characterise the significant biological motifs, structure, and complex architectures of common starting materials that dictate cell behaviour and tissue responses differentiating from disease phenotypes.^18^ Cells are sensitive to organisational motifs discussed above, such as fibril status,^19^ molecule length, and molecular weight^20^ as these may indicate tissue degradation and induce an inflammatory response. Finally, this review aims to highlight the importance of target tissue and substrate material characterisation, starting material selection and processing based on innate biologically active motifs, with final validation of retained biological motifs in the final product to regenerate the target tissue.

## Functions and bioactivities of natural polymers

**Fig. 1:**
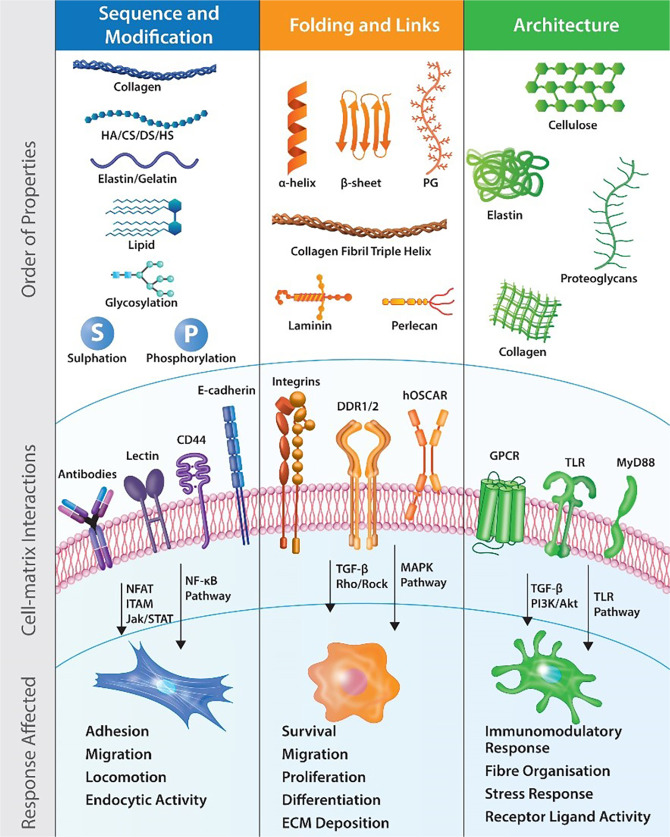
Examination of functional sites on macromolecules from sequentially based cell recognition to tertiary, 3-dimensional architectures that influence cell signalling. Cell surface receptors recognise material motifs and activate specific signalling cascades in response. CD44 cluster of differentiation 44, CS chondroitin sulphate, DDR1/2 discoidin domain receptors 1/2, DS dermatan sulfate, GPCR G-protein coupled receptor, HA hyaluronic acid, hOSCAR human osteoclast associated receptor, HS heparan sulphate, ITAM immunoreceptor tyrosine based activation motif, Jak/STAT Janus kinase/signal transducer and activator of transcription, MAPK mitogen-activated protein kinase, MyD88 myeloid differentiation primary response 88, NFAT nuclear factor of activated T-cells, NF-κB nuclear factor kappa-light-chain-enhancer of activated B cells, PI3K/Akt phosphatidylinositol 3-kinase/protein kinase B, PG proteoglycan, Rho/ROCK Rho/Rho-associated coiled-coil kinase, TGF-β transforming growth factor Beta, TLR toll-like receptor

Large macromolecules such as proteins (collagen, silk, elastin, gelatin, keratin, titin, fibrin, and mucin), polysaccharides (glycosaminoglycans, cellulose, methylcellulose, amylose, chitin, starch, dextran, agarose, and alginate), proteoglycans (aggrecan, versican, neurocan, and lumican), and nucleic acids (DNA/RNA) are used primarily to fulfil a physical function, as a scaffold, carrier, or substrate for functional modifications, however, these starting polymers possess intrinsic biological activity to enhance the bioactivity of the final material (Fig. 1). Further, these macromolecules are cross-linked heavily with non-natural cross-linking methods to add functionality. While the focus has moved to include these biological components in implantable biomaterials, implant design, material processing, and final production fail to take advantage of the multitude of biological functions these components possess. Innate motifs, domains and properties of natural polymers (non-mammalian and mammalian) have been outlined in Table 1.

**Table 1:** Summary of bioactive sequences, domains, and structures of commonly used natural materials for biomaterials

Macro-molecule	Molecule family	Binding Site/Functional Motif	Receptor	Activated signalling pathway	Downstream effects	Refs.
*Primary sequence*						
Collagen	Structural protein	GFOGER sequence (dependent on fibrillary status)	Integrins (α_1_β_1_, α_2_β_1_, α_10_β_1_, and α_11_β_1)_	Erk1/Erk2 (p44/42) mitogen-activated protein kinase pathway	Adhesion, migration, survival	^50^
		GPOGPX′GFX′ sequence	OSCAR	NFAT pathway via the CD3ζ cytoplasmic signalling domain.	Cardiac and skeletal development, inflammation and immune response	^58^
		GPO repeats	GPVI	ITAM (pathway, activates Syk proteins and tyrosine phosphorylation	Platelet tethering, activation, adhesion, aggregation, and degranulation	^59^
		Fragmented collagens, GPO repeats	G6b-B	Immunoreceptor tyrosine-based inhibition motif (ITIM)/ tyrosine-based switch motif (ITSM).	Phosphatase activation and inhibition of activated phosphorylated molecules, e.g., differentiation, immune function	^55,57^
		GPO repeats	LAIR-1 of Leukocyte receptor complex	SHP-1 and SHP-2 phosphatases activation		^30,38,52,53^
		Collagenase cleavage site	uPARAP/Endo180 (FN-II domain)	Binding and internalisation of collagen	Cell adhesion, migration, bone development, and ECM remodelling	^60,61^
		Glycosylated collagen	uPARAP/Endo180 (Lectin Domain)	Modulate the endocytic efficiency of uPARAP/Endo180		^61^
		Glutamine and lysine residues of adjacent fibres	Transglutaminase 2	Creates ε(γ-glutamyl) lysine cross-links to stabilise the 3D structure	3D structure, osteoclastic activity, and bone formation	^17,67^
		Mono-glycosylated (Gal-Hyl) and diglycosylated (Glc-Gal-Hyl)	Collagenases	Reduced digestion rates, increased thermal stability	Fibril formation, resistance to degradation	^62^
Gelatin	Hydrolysed form of collagen	GxOGER and RGD sequences	α_V_β_3_ and α_5_β_1_ integrins	RhoA–GTPase	Cytoskeletal organisation, cell adhesion, and movement	^70^
Laminin	Fibrous protein	Specific laminin isoforms	α_6_β_1_ and α_V_β_1_ integrins	Jak/ STAT-pathways, MAPK-pathways, and PI3K/Akt-pathways	Pluripotency and cell differentiation	^86^
Elastin	Fibrous protein	RKRK sequence	α_V_β_3_ integrin	MAPK-pathways and PI3K/Akt-pathways	Cell proliferation, migration, and angiogenesis	^83^
		GxxPG consensus sequence	Elastin receptor complex	MEK1/2/ERK1/2 pathway	Proliferation, differentiation, and tumorigenesis	^84^
Fibrin/fibrinogen	Insoluble fibrous protein	NH_2_-terminal regions of fibrin β chains	VE-cadherin/ VEGF receptor 2	Inhibits VEGF R2 phosphorylation and MAPK signalling	inflammation and angiogenesis	^301,302^
Silk fibroin and sericin	Insoluble fibrous protein	GSGAGA	RANKL	Inhibition of ERK1/2 signalling	Osteoclastogenesis	^43^
		VITTDSDGNE and NINDFDED sequences	α_5_β_1_ integrin	ERK 1/2 and JNK 1/2 kinases, c-Jun and c-Jun protein	Cell migration	^44,45^
Vitronectin	ECM glycoprotein	RGD sequence	α_V_β_3_ integrin	Focal adhesion kinase (FAK) and p-ERK	Cell proliferation, migration, and angiogenesis	^303,304^
Tenascin	ECM glycoprotein	VFDNFVLK	α_7_ and α_9_β_1_, α_V_β_3_ integrin	p38 MAPK signalling	MSC differentiation and migration	^87^
CSPGs	ECM proteo-glycan	CS domain	Annexin 6	Surface localisation and endocytosis	Cell–matrix interaction and adhesion	^31^
		CS domain	Chemokines	Inhibit inflammatory receptor activation	Anti-inflammatory effect	^305^
		CS domain	Receptor protein tyrosine phosphatase σ (PTPσ)	Inhibits CRMP2, APC, S6 kinase and CREB proteins	Inhibit axonal growth	^102,103^
		CS domain	Nogo receptors 1 and 3	Modulate RhoA pathway	Inhibit axonal growth and CNS axon regeneration	^104^
HSPGs	ECM proteo-glycan	HS domain	GTPases, (Cdc42, Rac1, RhoA, RhoG, and ARF6)	Endocytosis via a caveolin-dependent pathway	Endocytic activity	^113,114^
		HS domain	APRIL	The exact mechanism is unknown	Promote cell proliferation and tumour growth	^115^
		HS domain	VLDL	Internalisation, independent of LRP-1 endocytic activity	Regulate lipid metabolism	^116^
KSPGs	ECM proteo-glycan	KS chains—sulphation pattern	TLR4 receptor	Sulphation inhibits TLR4 activation and signalling	Anti-inflammatory effect reduced macrophage recruitment	^107,108^
		KS domain	SHH, FGF1, and FGF2	Regulate growth factor binding to receptors	Growth and protein synthesis	^110,111^
		KS domain	Robo-Slit	Rho family GTPases	Cytoskeletal organisation, cell movement	^107^
Decorin	ECM proteoglycan (CS/DS chains)	LRR6 domain	LRP-1	Smad 2/3/7 and PI3K activation	Modulate TGFβ signalling and cell proliferation	^96,306^
Lumican	ECM proteoglycan (KS chains) - SLRP	C-terminal	Transforming growth factor-β receptor 1	pERK1/2 signalling	Cell growth and differentiation	^98^
Versican	ECM proteoglycan	CS domains	L-selectin and P-selectin, CD44	Β-catenin and wnt signalling	Cell differentiation, locomotion, and inflammation	^90,91^
Hyaluronic acid	Glycosaminoglycan	HA disaccharide units of *N*-acetyl glucosamine and glucuronic acid	CD44	Rho and Rac1 GTPases	Migration and proliferation and cell–matrix interactions	^69,70,72–74^
			Receptor for HA-mediated motility (RHAMM)	Tyrosine kinase, pp60c-src	Cell locomotion	^75^
HA/CS/HS	Glycosaminoglycans	Repeating disaccharide	Anti-DNA antibodies	MHC macrophage activation	Inflammatory response	^105,106^
GAGs	Glycosaminoglycans	GAG sequence	PRELP	Inhibition of NF-κB transcription	Differentiation, inflammation	^101^
Chitin/Chitosan	Polysaccharide	Chitin and peptidoglycan	RegIIIA, a secreted C-type lectin	STAT3 tyrosine phosphorylation	Cell proliferation or differentiation, anti-apoptotic	^26^
		β-galactosides	Galectin-3	ERK, AKT, and JAK/STAT1 pathways	Immune response	^27,28^
*Secondary and tertiary property recognition (MW, sequence length, domain conformation, and fragmentation)*						
Collagen	Structural protein			RHOA-ROCK signalling pathway	Cytoskeletal organisation, mechanosensing, and cellular contraction	^307^
		Fibrillar collagens I–III (must be intact)	Discoidin domain receptors DDR1 and 2	Tyrosine auto-phosphorylation and receptor internalisation	Proliferation, migration, survival	^64–66^
		Phosphorylation/dephosphorylation stabilise triple helical structure	Collagenases and uPARAP/Endo180 (FN-II domain) activation	Destabilised non-conforming triple-helical orientation are subject to degradation	Synthesis, assembly, signalling, and immune response	^63^
		Requires procollagen structure. Binding sequence: xGxR	Hsp47	Hsp47 binds to procollagen in the ER and dissociates in the cis-Golgi inhibiting the aggregation of collagen for a proper triple helix assembly	Facilitate triple helix formation	^308,309^
Elastin	Fibrous protein	Mature elastin matrix	G-protein-coupled receptors	Rho-mediated signal transduction pathway	Actin stress fibre organization, proliferation, and migration	^85^
HA	GAG	LMW fragmentation products of HA	TLR4	Phosphorylation of p38/p42/p44 MAP-kinases and NF-κB	Cell differentiation and inflammation	^80,81^
DS	GAG	DS fragments (octa-saccharides and hexa-saccharides	Heparin cofactor II	Serine proteinase inhibitor	Inhibits coagulation	^310^
Mucin	Polysaccharide	Extracellular α-chain of transmembrane mucin domain	Cell-surface receptors	Barrier function, receptor shielding	Immune response modulation and cell–cell contact	^311^
Cellulose	Polysaccharide	Carbohydrate oligomers and polymers	TLR/MyD88 dependent	TLR2-mediated NF-κB activation	Immunomodulatory effects	^37^
Chitin/chitosan	Polysaccharide	Carbohydrate oligomers and polymers	TLR/MyD88 dependent	Secretion of cytokines and chemokines	Immunomodulatory effects	^29^
		Chitin and chitin fragments	FIBCD1, type II transmembrane protein	IL-4 and IL-13 mediated MAPK activation, reactive oxygen species generation	Immune response modulation	^30^
		GlcNAc and linear chitooligomers, chitotetraose	NKR-P1	FcRγ ITAM residues and downstream recruitment of Syk tyrosine kinase	Immune response, NK cytotoxicity	^31^
Heparan sulphate	GAG	HS chains	Sclerostin	Negatively regulates Wnt/β-catenin signalling pathway	Regulation of bone formation and homeostasis	^312^
Fibromodulin and lumican (KS)	GAG	Collagen helix cross-linking sites; putative KGHR binding sequence	Collagen	Regulate fibrillogenesis	Fibromodulin promoting the formation of thick fibres while lumican promotes the formation of thin collagen fibres	^313^
Keratan sulphate	GAG	Sulphated chains—degree of sulphation	Slits protein family	Activation of Rho GTPases	Actin depolymerisation, cytoskeletal reorganization	^107^
Versican	Proteoglycan	Versican G1 domain, HABR domains, Ig-like domain	Hyaluronan	Leukocyte binding, versican binding HA affects HA binding to CD44	Cellular behaviour, inflammation	^314^
Laminin-332/511/211/221/111	Fibrous protein	LG1-3 domain cluster	Integrin α_3_β_1_, α_7_β_1_, α_6_β_1_, α_6_β_4_	Rho-GTPases Cdc42 and Rac1 activation. Phosphorylation of specific tyrosine residue of the β4 cytoplasmic domain	Cell polarization, migration, and survival, cell-matrix complexes, basement membrane assembly, growth factor receptors’ signalling	^315–317^
		LG1-3 domain cluster	Integrin α_6_β_4_			^315,317^
Laminin-332	Fibrous protein	Heparin-binding domain located in the LG4-5 modules	Syndecan-4	β1 integrin activation	Attachment of fibroblasts, endothelial cells, and keratinocyte migration	^315,318,319^
SLRPs	ECM proteoglycans	Leucine-rich repeats (LRR) domain	Collagen	Regulates lysyl oxidase activity on lysine residues for collagen assembly	Regulate collagen fibrillogenesis, fibril diameter, spacing, and patterning	^95,320^
HA	GAG	High molecular weight HA molecule	CD44	Blocking cytokine signalling	Anti-inflammatory properties	^321,322^
Perlecan	PG	Protein core or HS chains	FGF2	Protect several growth factors from degradation and misfolding	Cell proliferation, differentiation, and angiogenesis	^323^
		Domain III protein core	PDGF			^323^
		Domain V	FGF7			^323^
		Domain IV and V	Heparin			^323–325^
		Domain I	Thrombospondin	Receptor-mediated endocytosis	Regulation of cell adhesion, migration, and proliferation	^323,326^
Versican	PG	PTR (proteoglycan tandem repeats)	Hyaluronan	Formation of ternary complexes modulates division and migration	Facilitate cell proliferation and migration	^327,328^

Biologically active sequences may be sub-classified into primary sequence recognition sites (in which a receptor directly bind with a specific amino acid sequence or carbohydrate repeats) and secondary and tertiary structures (which depend on molecular conformation, motifs, and domains for recognition). Biomaterials contain naturally occurring functional sequences and motifs that bind receptors to induce intracellular signalling and promote downstream effects

*APC* adenomatous polyposis coli, *APRIL* a proliferation-inducing ligand, *ARF6* ADP ribosylation factor 6, *Cdc42* cell division cycle 42, *CD3ζ* cluster of differentiation 3ζ, *CRMP2* collapsin response mediator protein 2, *CREB* cAMP-response element binding protein, *CSPGs* chondrotin sulphate proteoglycans, *DDR* discoidin domain receptor, *Erk* extracellular signal-regulated kinase, *ECM* extra-cellular matrix, *FAK* focal adhesion kinase, *FGF* foetal growth factor, *GAG* glycosaminoglycan, *GPVI* immunoglobulin (Ig) superfamily member glycoprotein VI, *GTPase* guanisone diphosphotase, *Hsp47* heat shock protein 47, *HSPGs* heparan sulphate proteoglycans, *ITAM* immunoreceptor tyrosine-based activation motif, *ITSM* immunoreceptor tyrosine-based switching motif, *Jak/STAT-* Janus kinase/signal transducer and activator of transcription, *KSPGs* keratan sulphate proteoglycans, *LRP-1* lipoprotein receptor-related protein 1, *LMW* low molecular weight, *LRP-1* lipoprotein receptor-related protein 1, *MAPK* mitogen-activated protein kinase, *MHC* major histocompatibility complex, *MSC* mesenchymal stem cells, *NFAT* nuclear factor of activated T-cells, *NF-κB* nuclear factor kappa-light-chain-enhancer of activated B cells, *OSCAR* osteoclast associated receptor, *PDGF* platelet derived growth factor, *PI3K* phosphoinositide 3-kinases, *PRELP* proline/arginine-rich end leucine-rich repeat protein, *PTPσ* protein tyrosine phosphatase σ, *RANKL* receptor activator of nuclear factor-κB ligand, *Rac1* Ras-related C3 botulinum toxin substrate 1, *RHAMM* receptor for HA-mediated motility, *RegIIIA* regenerating islet-derived protein 3 alpha, *RHOA-ROCK* Rho/Rho-associated coiled-coil containing protein kinase, *CD3ζ* cluster of differentiation 3ζ, ITAM, *SHP* Src homolog phosphatase, *SHH* Sonic HedgeHog, *Smad* Sma mothers against decapentaplegic homolog, *SLRP* small leucine rich proteins, *TLR* toll-like receptor, *TGF-β* transforming growth factor beta

### Non-mammalian polymers

Non-mammalian natural polymers are in high abundance and easily sourced. These polymers that include chitosan, alginate, and dextran are easily extracted and purified from plant sources yielding a low-cost material with low antigenicity.^21,22^ Chitosan, a water-soluble derivative of chitin is a natural polysaccharide made of glucosamine and a fraction of *N*-acetyl-glucosamine.^23^ This biodegradable, non-toxic polymer can be obtained by processing chitin extracted from crustaceans through a deacetylation process.^24^ Chitosan possesses the highest chelating ability of all-natural polymers, a vital detoxification property.^25^ Chitin and chitosan interact with regenerating islet-derived protein 3-alpha (RegIIIA), a secreted C-type lectin (formerly HIP/PAP) induce STAT3 tyrosine phosphorylation and stimulate interleukin-22 and interleukin-6 secretion.^26^ Galectin-3 binds to β-galactosides of chitin to activate extracellular-signal-regulated kinase (ERK), protein kinase B (AKT), and Janus kinase/signal transducer and activator of transcription protein (JAK/STAT1) signalling pathways, resulting in a dysregulated release of pro-inflammatory cytokines.^27,28^ Chitin and chitosan oligomers and fragments activate toll-like receptor/myeloid differentiation primary response 88 (TLR/MyD88), fibrinogen C domain-containing protein 1 (FIBCD1), and NK cell receptor protein 1 (NKR-P1) to upregulate cytokines and chemokines and activate natural killer (NK) cytotoxicity.^29–31^ Chitosan has been shown to induce IL-10 secretion into animal blood and suppress colitis, through the modulation of nuclear factor kappa-B (NF-κB) signalling.^32,33^ Overall, chitin induces cell proliferation and differentiation, while also propagating an immunomodulatory effect.^34^ While chitin and chitosan are attractive materials due to their availability and biodegradability, pro-inflammatory signalling may be a drawback for widespread use, especially in degenerative disease with upregulated inflammatory cascades.

Alginate is a naturally occurring polysaccharide found in seaweed commonly used in drug delivery devices. Agarose is a polysaccharide, comprised of a basic repeat unit consisting of 1,3-linked-d-galactopyranose and 1,4-linked 3,6-anhydrous-α-l-galactopyranose, derived from algae.^35^ It is often used as a matrix to encapsulate cells and undergoes thermal cross-linking.^36^ Methylcellulose is a non-toxic polysaccharide derived from cellulose that undergoes gelation at 37 °C. Cellulose, methylcellulose, and agarose are all also used commonly in the biomedical industry. Cellulose does induce toll-like receptor (TLR)2-mediated NF-κB activation, promoting immunomodulatory responses,^37^ however, these materials are mainly considered inert and suitable for in vitro assays. The limitation of using these materials is that mammalian cells lack receptors to bind to these plant-based polysaccharides, and do not produce catabolic enzymes to degrade them. This lack of cell–matrix interaction will influence gene expression in cultured cells, altering cell phenotype.^38^

Dextran is a neutral, biodegradable polysaccharide with an α-(1,6) pyranose ring linkage, formed from sucrose by dextransucrose enzyme by different bacterial strains. These properties make it suitable for use as scaffolds in tissue engineering.^39^ Dextran has been shown to increase cell adhesion and proliferation and upregulates gene expression of endothelial markers.^40^ These effects were mediated by upregulation of phosphoinositide 3-kinases (PI3K/Akt), ERK1/2, c-Jun N-terminal kinase (JNK), and p38 mitogen-activated protein kinases pathways in response to dextran.^40^ Dextran has been evaluated to demonstrate antioxidant, anticoagulant, and immunomodulatory properties through physical properties and the activation of pathways mentioned above, however, its use is limited by its mechanical properties when used in isolation.^39^

Silk is a natural fibre obtained from silkworms (*Bombyx mori*) and spiders (*Nephila clavipes*).^41^ Silk fibroin is a fibrous protein consisting of 17 amino acids that, together with Sericin, makes up a silk fibre. This material does not induce adverse effects in implanted tissue. It has been widely used in skin closure sutures due to its tensile strength. Functional differences have been characterised in among silks originating from different species due to heterogeneity in amino acid sequence.^42^ Silk fibroin binds to receptor activation of nuclear factor κB ligand (RANKL) causing ERK1/2 signalling and expression of NF-κBp65, which promotes induction of osteoclastogenesis.^43^ Fibroin and Sericin are recognised by α_5_β_1_ integrin at VITTDSDGNE and NINDFDED peptide sequences to upregulate of c-Jun and c-Jun protein phosphorylation. Moreover, fibroin and sericin stimulate phosphorylation of ERK 1/2 and JNK 1/2 kinases.^44,45^ Silk fibre proteins, fibroin and sericin, activate intracellular pathways that are immunomodulatory and induce cell proliferation and differentiation.

Non-mammalian polymers maintain a prominent role in tissue engineering given their abundance, relative ease of isolation, and proven efficacy. The application of these materials in implantable devices warrants further consideration given the pro-immunogenic responses recorded in several studies, though this may be initiated by low isolation and antigen removal. The prominence of these materials is unlikely to diminish significantly given the role of silk products in wound healing, for example, while further integration to regenerate tissues and organs internally is less promising.

### Mammalian polymers

The use of mammalian polymers can be advantageous over non-mammalian counterparts due to their enhanced biocompatibility and biodegradability, although potentially limited by their inherently low mechanical properties when unmodified or disorganised.^46^ While their isolation is more complicated than non-mammalian substrates, their biological activity through receptor recognition sites and conformational changes prompt further research into their use. These polymers include proteins such as collagen, gelatin and elastin, polysaccharides such as hyaluronic acid, heparan sulphate, and chondroitin sulphate and combinations in the form of glycoproteins and proteoglycans.

Collagen is a highly suitable natural polymer for tissue engineering purposes, considering it is the main constituent of the ECM that promotes cell proliferation and tissue formation due to its mechanical and biological properties. Collagen plays a crucial role in maintaining the structural integrity and spatial organisation of the ECM and is involved in essential cell activities such as morphogenesis, ECM deposition, tissue repair, and remodelling.^47^ Collagen-based implantable materials are fabricated using purified collagen solutions, isolated from animal tissues such as skin, tendon, or articular cartilage and can be discussed in two distinct categories; hydrogels made up of collagen fibres that may be further modified and decellularised tissues with complete collagen architecture.^48^ Decellularised matrices arise from whole tissues that are treated to remove cells and immunogenic antigens, retaining the original ECM organisation of the tissue with functional proteins intact.^49^ Both of these approaches to using collagen as a scaffold have advantages and disadvantages when considering the bioactivity of the final matrices.

The bioactivity of collagen has been studied in detail, and its peptide sequences induce intracellular signalling through many pathways. Herein, we summarise the many roles of collagen in cell–material interactions. Many collagen-sequence specific receptors have been characterised to date. Several integrins (α_1_β_1_, α_2_β_1_, α_10_β_1_, and α_11_β_1_) bind to the GFOGER peptide sequence of collagen activating the Erk1/Erk2 (p44/42) mitogen-activated protein kinase signal transduction pathway to promote cell survival, adhesion, and migration.^50^ Upon ligand binding, integrins undergo conformational changes to induce “outside-in” signalling. This activates multiple signalling events that differ across cell populations and are dependent on other signalling receptor activation.

Gly-Pro-Hyp (GPO) repeats are recognised collagen ligand binding sites, crucial for fibril alignment essential to protect against external stresses on the tissue.^51^ These GPO repeats are subject to glycation, increasing the stiffness of the tissue.^51^ Leukocyte-associated Ig-like receptor (LAIR-1) and G6b-B receptors, expressed on megakaryocytes and platelets, are specific for GPO repeats and act to inhibit differentiation of immune cells.^52^ LAIR-1 is present during osteoclastogenesis and inhibits bone remodelling.^53^ LAIR-1 contains two immunoreceptor tyrosine-based inhibition motifs (ITIMs), which when phosphorylated, recruit Src homology phosphatase 1 (SHP-1) and SHP-2. These phosphatases directly dephosphorylate Syk, Zap70, and PLCγ, preventing immunoreceptor tyrosine-based activation motif (ITAM)-mediated stimulation of protein kinases and calcium signalling.^30,38^ G6b-B, an inhibitory platelet receptor, is activated by collagen fragments, such as in response to the recognition of damaged epithelium.^54,55^ G6b-B contains one ITIM and an immunoreceptor tyrosine-based switch motif (ITSM),^55^ which in contrast to ITIM (directly signals through activation of phosphatases), interferes with ITAM activity through adaptor molecules.^56^ In megakaryocytes and platelets, G6b-B interferes with ITAM-mediated signalling induced by collagen binding to Immunoglobulin (Ig) superfamily member Glycoprotein VI (GPVI).^57^

Collagen activates stimulatory receptors such as osteoclast associated receptor (OSCAR) and GPVI. OSCAR is specific to the GPOGPX′GFX′ sequence of the triple-helical peptide. It induces nuclear factor of activated T-cells (NFAT) signalling via the CD3ζ cytoplasmic signalling domain to induce osteoclast differentiation, and sustained function^58^ GPVI is located on platelet surfaces and binds to collagen fragments in coagulation.^59^ Collagen binds to OSCAR or GPVI to recruit ITAM-containing FcR-γ chains. OSCAR activation causes the initiation of calcium signalling, which is necessary for the activation of NFAT c1, and osteoclastogenic transcription factor. Activated GPVI binds Syk to the FcR-γ chain, which in turn activates Syk proteins and tyrosine phosphorylation. These interactions are dependent on the intact fibrillar conformation of collagen to activate downstream signalling for osteoclastogenesis and coagulation, respectively.

GPVI is also specific for GPO repeats on collagen, activating platelet adhesion and aggregation through inside-out signalling of integrins.^59^ Urokinase plasminogen activator receptor-associated protein (uPARAP/Endo180, FN-II domain) is specific for the collagenase cleavage site, GXY triplets, which may be masked by the triple-helical conformation of native collagen. When the collagen triple helix unfolds during degradation, these motifs are recognised and internalised. This process plays a role in tissue remodelling and bone development.^60,61^ The lectin domain of uPARAP/Endo180 binds to glycosylated motifs on collagen, modulating the endocytic efficiency of the receptor towards favouring highly glycosylated collagens such as basement membrane collagen IV.^61^ Glycosylation motif distribution on collagen also affects digestion rates by mammalian collagenase, where mono-glycosylated (Gal-Hyl) and diglycosylated (Glc-Gal-Hyl) reduced enzyme activity and increased thermal stability of collagen.^62^ Thus, sourced collagen as a starting material may be hyper-glycosylated or hypo-glycosylated to tune the biomaterial towards desired properties for its intended application.

Triple helical formation of collagen peptides stabilises formed fibrils, providing characteristic mechanical properties. Phosphorylation of collagen peptide is a significant modification that stabilises the triple helix independent of sequence.^63^ Phosphorylation/dephosphorylation acts as a stabilisation switch, changing melting temperature by up to 13 °C, effecting synthesis assembly and signalling.^63^ The Discoidin domain receptors 1 and 2 (DDR1/2) recognise intact collagen I, II, and III fibrils activating tyrosine auto-phosphorylation with a unique activation pathway, to induce receptor internalisation.^64^ Activation of DDRs promotes cell survival, proliferation and migration.^65,66^ Dysregulation of DDR1 is involved in the development of fibrosis, atherosclerosis, arthritis, and cancer.^64^

Collagen architecture is formed by fibrils and fibre formation and transglutaminase induced crosslinks formed by linking glutamine and lysine residues to reinforce the three-dimensional structure.^67^ These crosslinked collagen fibrils act as a substrate for osteoclasts and bone mineralisation.^17^ On a supramolecular level, collagen binding of growth factors such as transforming growth factor-β (TGFβ) modulates the ligand-receptor activity increasing TGFβ signalling.^68^ Synthesis and organised deposition of collagen fibres increases ECM stiffness and promotes an intracellular actomyosin network organisation in epithelial cells. This mechanosensing process mediated by the activation of Rho-associated kinase (RHOA-ROCK) signalling pathway to initiate cell contraction. The importance of collagen on branching morphogenesis and tissue formation is apparent, when collagenase treatment completely inhibits submandibular gland cleft formation and branching.^69^ Collagen may be the further process to form gelatin, synthetic colloids made of denatured collagen polypeptides, a product of bovine cartilage degradation. This synthetic polymer retains the GxOGER and RGD sequences, sensed by α_v_β_3_ and α_5_β_1_ integrins to activate ERK signalling and regulate cell adhesion and mechanosensing.^70^ Collagen exerts a multitude of biological activities through numerous receptor interactions and signalling cascades that are sequence, conformation, PTM, and organisationally dependent. Thus, there is a broad scope for tuning collagen based starting materials through manipulation of the base sequence, PTM coverage, fibril formation, and cross-linking to create the final product with the desired biological activities. Collagen has been used in hydrogel blends to increase cellular interaction and attachment. 3D hydrogel network of collagen/alginate exhibited uniform distribution of collagen fibrils and maintained neural progenitor cell attachment via α_1_β_1_, α_2_β_1_ integrin.^71^ Adherent neurons on collagen fibrils within the 3D hydrogel matrix promoted the maturation and formation of neural networks. Collagen may be modified with carbohydrate residues to mimic natural PTMs to differentially influence the glyco-signature of primary neuronal cells.

Hyaluronan is a simple, non-sulphated glycosaminoglycan that is a significant constituent of the ECM. Hyaluronan is a crucial component in the pericellular matrices of migrating cells in the developing embryo, in proliferating cells in regenerating tissues and in other dynamic cellular events. This hydrated pericellular substrate interacts with proteoglycans and other extracellular macromolecules to form a template that facilitates cell migration, and the assembly of ECM and pericellular matrices.^72^ Hyaluronan interacts with cell surface receptors, such as receptor for HA-mediated motility (RHAMM) and cluster of differentiation-44 (CD44).^72^ CD44 and RHAMM both contain a B(X7)B sequence where B is an essential amino acid residue, arginine or lysine, and the X’s contain at least one essential amino acid but can be any other non-acidic amino acids, which will bind to the HA disaccharide units of *N*-acetyl glucosamine and glucuronic acid.^73,74^ In morphogenesis, interactions between hyaluronan with the cell surface receptors RHAMM and CD44 are involved in cell locomotion and proliferation, which are critical events in morphogenesis.^73,75^ HA–CD44 interactions mediate endocytic removal of HA, a critical regulatory process in essential stages of embryonic development. Hyaluronan–CD44 signalling can activate Rho and Rac1 GTPases, leading to reorganisation of the actin cytoskeleton^76^ erbB2 tyrosine kinase activation,^77^ and cell proliferation through src-related tyrosine kinases,^78^ and NF-κB.^79^ RHAMM activation induces a protein tyrosine kinase signal transduction pathway (pp60c-src) causing downstream activation, that modulates focal adhesions for RHAMM-associated mediated cell motility.^75^ High molecular weight (HMW) HA has been shown to promote tumorigenesis, antiangiogenic and anti-inflammatory responses in breast-cancer cell lines while low molecular weight (LMW) HA is implicated in cell motility, CD44 cleavage, and angiogenesis. LMW fragmentation products of HA induce phosphorylation of p38/p42/p44 MAP-kinases and nuclear translocation of NF-κB, which are components of the TLR-4 signalling pathway, a pro-inflammatory signalling pathway.^80,81^ Thus, the molecular weight of the HA ligand is crucial for its recognition and biological function.^82^ The biological activity of HA is well characterised, especially concerning the influence of MW on immunomodulatory effects. Biomaterial design using HA as a starting material must consider the molecular weight of HA, cross-linking method for receptor binding and degradation and fragmentation products to ensure suitability for the final application, promote an anti-inflammatory response and to mitigate adverse effects.

Elastin is a crucial ECM protein that provides elasticity and integrity to tissues. Elastin binds α_V_β_3_ integrins at the RKRK sequence promoting cytoskeletal assembly and cell adhesion.^83^ Elastin is also bound by Elastin receptor complex at GxxPG consensus sequences, which activates the ERK1/2 pathway through a signal dependent on protein kinase A and phosphoinositide 3-kinase c (PI3Kc), regulating pro-tumoral signalling.^84^ The mature elastin matrix triggers G-protein coupled receptor-mediated Rho signalling pathway to induce actin-stress fibre organisation, inhibit proliferation, and regulate migration.^85^ Maintenance of the RKRK sequence of elastin and availability in the final product is essential for cell integration and organisation in the material.

Peptide sequence availability is also necessary for Laminins, ECM proteins found mostly in the basal lamina of the basement membrane, to activate signalling cascades. Laminin isoforms are ligands for α_6_β_1_ and α_V_β_1_ integrins, which activate Jak/STAT-pathways, MAPK-pathways, and PI3K/Akt-pathways. These processes influence cell renewal, differentiation, and promote retained expression of pluripotency markers.^86^ The tenascin specific sequence, VFDNFVLK, is recognised by α_7_ and α_9_β_1_, α_V_β_3_ integrin to activate p38 MAPK signalling.^87^ This interaction has been shown to affect neuronal differentiation and mesenchymal stem cell (MSC) migration.^87^ The tenascin specific sequence may be preserved or blocked, through targeted cross-linking, depending on desired material function.

Proteoglycans (PGs), commonly used in tissue engineering, are glycosylated proteins typically found in the ECM, anchored to HA. Aggregating PGs, such as aggrecan and versican, aggregate on HA via link protein interactions. Aggrecan is the most glycosylated PG, consisting of up to 30 keratan sulphate (KS) chains and 150 chondroitin sulphate (CS) chains, and is highly abundant in cartilage.^88^ These glycosaminoglycan (GAG) chains form negatively charged aggregates that efficiently retain water and resist compression and deformation.^89^ Versican is a large interstitial PG with many chondroitin sulphate chains with an apparent MW of more than 1000 kDa.^89^ Versican is bound by leukocyte adhesion molecules L-selectin and P-selectin on its CS domains, which may only occur depending on sulphation pattern.^90,91^ The carbohydrate-binding domain of CD44 also recognises the CS chains of versican. Versican can induce integrin and epidermal growth factor receptor (EGFR) activation to initiate neuronal cell differentiation.^92^ Versican also has an ability to bind chemokines, downregulating their function.^93^ Recently, different isoforms of versican have been shown to induce opposite effects on cell proliferation, likely through competitive binding of CD44 with HA.^94^

Nonaggregating PGs, such as small leucine-rich PGs (SLRPs), consist of relatively smaller protein cores with fewer GAG chains than aggregating PGs. SLRPs including decorin, biglycan, lumican, fibromodulin, and proline arginine-rich end leucine-rich repeat protein (PRELP) all interact heavily with cell-surface receptors.^95^ Decorin, the most abundant SLRP in cartilage, binds to low-density lipoprotein receptor-related protein 1 (LRP-1) and causes internalisation of the complex, modulating of TGFβ signalling via Smad 2/3/7 and PI3K activation.^96^ Decorin also binds EGFR, triggering dimerization, endocytosis and degradation of the decorin-receptor complex, presumably inhibiting signalling.^97^ Lumican interacts with Transforming growth factor-β receptor 1 (ALK5) activating pERK1/2 signalling to promote wound healing.^98^ Fibromodulin, decorin, asporin, and biglycan both bind TGFβ and other growth factors and potentially inhibit activity.^99,100^ The glycosaminoglycan-binding domain of PRELP acts as a cell type-specific NF-κB inhibitor via CS-annexin II-dependent internalisation to impairs osteoclastogenesis.^101^ SLRPs are integral to the structural architecture of connective tissues through the binding collagens, ECM glycoproteins, and other extracellular and membrane bound components. Decorin, lumican, fibromodulin, PRELP, asporin, and chondroadherin all regulate collagen fibrillogenesis, to determine fibril diameter, spacing, and patterning.^95^

The above-discussed PGs contain GAG chains with many functions. These have been shown to assert biological activity on their own or in aggregation with PGs. Chondroitin sulfate in CS proteoglycans (CSPG) is recognised by annexin-6, aiding in cell surface localisation and cell adhesion.^31^ Receptor protein tyrosine phosphatase σ (PTPσ), on the first immunoglobulin-like domain, and its subfamily member LAR act as transmembrane receptors that mediate growth inhibition of CSPGs activating signalling pathways (RhoA, Akt, and Erk).^102,103^ Activation of PTPσ by CSPGs selectively inactivated collapsin response mediator protein (CRMP2), APC, S6 kinase, and cAMP response element-binding protein (CREB).

In contrast, leukocyte common antigen-related (LAR) phosphatase activation inactivated PKCζ, cofilin and liver kinase b1 (LKB1) and deletion of both receptors exhibit additive enhancement of axon growth in adult neuronal cultures in vitro.^102^ CSPGs also bind two receptors for myelin-associated growth inhibitors, Nogo receptors 1 and 3, where blockade of these receptors significantly increases CSPG signalling and promotes central nervous system (CNS) axon regeneration.^104^ HA, Chondroitin sulfate and heparan sulfate can capture anti-DNA antibodies due to a negative charge interaction, modulating the activity of these antibodies and possibly inducing an inflammatory response.^105,106^

Many keratan sulfate proteoglycan (KSPG)-cell receptor interactions have been characterised.^107^ Macrophages adhere to lumican core protein and spread rapidly; however, this process is inhibited by intact KS chains. The highly sulphated KSPGs of cornea inhibit macrophage adhesion; however, lumican desulphation occurs in corneal disease and localises macrophages to inflamed tissue.^108^ KSPGs also act as a barrier to neurite outgrowth in vitro and direct axon growth during development and regeneration in vivo.^109^ KS chains bind to insulin-like growth factor binding protein-2 (IGFBP2)^110^ and interact with sonic hedgehog (SHH), FGF1, and FGF2.^111^ KS disaccharides activate Robo-slit to induce Rho GTPase activity. This mediates actin depolymerisation, cytoskeletal reorganisation and cell signalling.^107^

Heparan sulphate, and HSPGs (syndecan, perlecan, and glypicans) cell–matrix interactions mediate endocytosis via caveolin-mediated pathways or lipid raft-mediated pathways.^112^ Small GTPases, such as Cdc42, Rac1, RhoA, RhoG, and ARF6 as well as actin also regulate HSPG internalisation.^113,114^ HSPG serves as a receptor for APRIL, a proliferation-inducing ligand, which is a member of the tumour necrosis factor (TNF) family, to promote cell proliferation and tumour growth.^115^ Very-low density lipoprotein (VLDL) binding and internalisation is dependent on complexing with cell-surface HSPGs. LRP-1 regulates HSPG expression through complexing, thus controlling the availability of VLDL binding sites at the cell surface. Upon dissociation from LRP-1, HSPGs bind to and internalise VLDL independent of LRP-1 endocytic activity.^116^

**Fig. 2:**
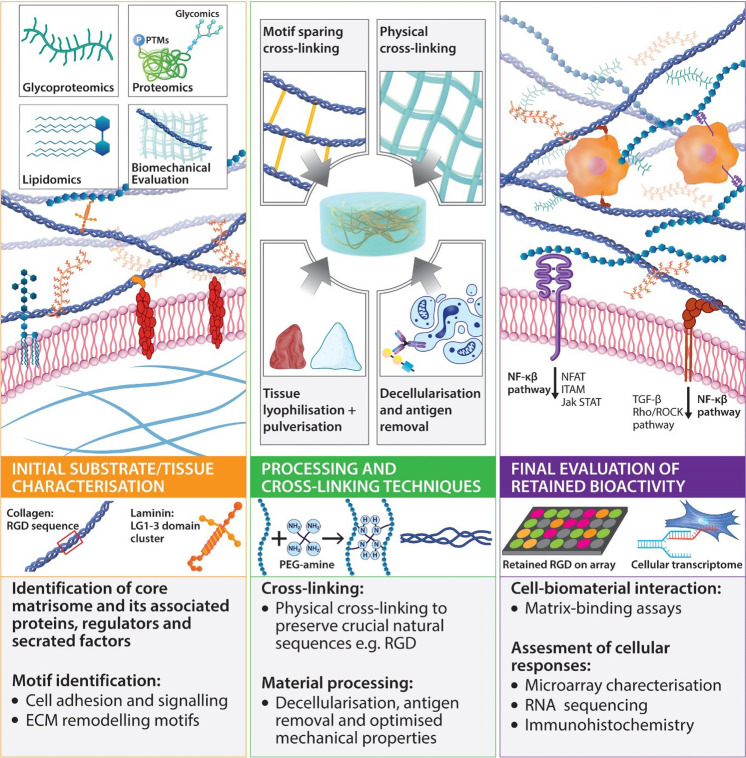
Summary of material design and processing methods that retain bioactivity. The first panel describes initial material characterisation to reveal crucial motifs present within the native tissue or starting material. Middle panel: further physical and chemical cross-linking must aim to conserve natural properties, avoiding modification of active motifs and sequences. The secondary structural organisation must be considered when modifying a starting material, and final products should mimic the mature network of the target tissue. Isolation and processing may aim to retain functional motifs associated with natural PTMs, while tissue processing and decellularisation techniques conserve many biological functions. PTMs post-translational modifications. Third panel: final evaluation is performed using functional assays and cell–matrix interaction arrays to confirm retained biological activity. ITAM immunoreceptor tyrosine-based activation motif, Jak/STAT- Janus kinase/signal transducer and activator of transcription, MAPK mitogen-activated protein kinase, NFAT nuclear factor of activated T-cells, NF-κB nuclear factor kappa-light-chain-enhancer of activated B cells, PTMs post-translational modifications, RHOA-ROCK Rho/Rho-associated coiled-coil containing protein kinase, CD3ζ cluster of differentiation 3ζ, TGF-β transforming growth factor Beta

ECM proteoglycans play an essential regulatory role in the organisation, degradation and cell signalling. The native properties and tissue-specific composition of the ECM provide an inherent biological activity to promote cell adhesion, proliferation, and differentiation.^95^ The ECM also acts as a reservoir of growth factors regulating protein synthesis and cell proliferation. The remodelling of ECM components induced by matrix metalloproteinases (MMPs) is a crucial process to allow cell migration and integration. Overall, ECM based implants act as dynamic and supportive scaffolds affecting cell migration, and differentiation, highlighting the role of cell–ECM interactions and ECM motifs as key stem cell determinants. Material processing and implant design must consider the above cell–matrix interactions to fulfil the starting material’s bioactive potential. This overview of cell–matrix interactions also highlights the variability of motif expression in different tissues and development, physiology, and disease. The starting material may consist of a purified protein, polysaccharide, or glycoprotein or be comprised of hundreds of constituents if derived from whole tissue products. We have highlighted the copious cell–material interactions that have been described in the literature with the most common starting materials. With material design trending towards more sophisticated and composite devices, we underscore the critical considerations to be made for innate material activity and further processing to modulate the activity of the final product to fulfil its application (proposed workflow described in Fig. 2).

## Characterisation of the initial target tissue and starting material

There has also been an increasing understanding of the role of abundant proteins, polysaccharides, and link proteins in modulating cell growth and tissue organisation. These molecules can also be highly modified with PTMs, including sulphation, phosphorylation, and glycosylation, which effect properties and function, necessitating characterisation to match the desired properties of the target tissue. Therefore, the initial characterisation of tissues and starting materials (Table 2) can inform device design to incorporate a combination of polymers with suitable modifications based on tissue characterisation to recapitulate the structure and function of the target microenvironment.

**Table 2:** Methods for initial molecular characterisation of starting materials and target tissues

Process	Target molecules	Process description	Outputs	Biological/design relevance	Refs.
Proteomics	Complete proteome (or specifically matrisome, secretome or intracellular)	MS/MS, LC-MS/MS-based analyses	Complete proteomic profile, with relative or absolute quantification dependent on the methodology	Identification key regulatory proteins to elucidate markers and molecular cues for tissue homeostasis and regeneration	^120,123,329,330^
Glycomics	Glycoproteins, *N*-glycans and *O*-glycans	*N*-glycosidase F/β-elimination, purification and detection using HPLC and MS/ MALDI-IMS	Complete *N*-glycan/ *O*-glycan profile. Localisation and functional analyses in purified proteins in mAbs. Spatial resolution in tissue sources	Complete *N*-glycome mapping of starting materials and target tissues. *N-*/*O*-glycosylation inform protein function, activity, and homeostatic status of tissues (including antigenicity)	^131,331,332^
Glyco-proteomics analyses	decorin, neurocan, brevican, aggrecan	GAG-linked peptides for each proteoglycan enrichment with LC-MS analyses	Glycoproteomics profile of proteoglycans	Proteoglycan glycosylation and characterization of glycosylated regions with domain localisation in different tissues and diseases	^333^
Lipidomics Analyses	Glycosphingolipids, LDL, HDL, VLDL, cholesterol	LC-ESI-MS/MS and MALDI-IMS	Characterization of the types, amounts, and tissue localization of sulphatides and other lipid modifications	Lipids can act as signalling molecules, which play a role in cellular fate—characterization of lipidomic profile for biomarker discovery and material functionalisation	^124,334,335^
PTM characterisation	ECM and site-specific collagen PTMs	HPLC and semi-quantitative, site-specific analysis by HPLC-tandem MS	Characterised collagen PTMs - Glycosylation	Tissues present distinct phosphorylation, acetylation, ubiquitination, methylation and *O*-GlcNAc patterns. Altered PTM patterning has regulatory role tissue homeostasis and cell phenotype and differentiation	^336^

*MS/MS* tandem mass spectrometry, *LC-MS* liquid chromatography-mass spectrometry, *HPLC* high-performance liquid chromatography, *MALDI-IMS* matrix-assisted laser desorption/ionisation imaging mass spectrometry, *mAbs* monoclonal antibodies, *LDL* low-density lipoprotein, *HDL* high-density lipoprotein, *VLDL* very-low density lipoprotein

Variation in the ECM composition of tissues can depend on age, gender, ethnicity, disease, lifestyle habits, and most prominently developmental stage.^117^ To design a device that reflects the structure and composition of the native tissue, complete, and rigorous profiling of the target tissue must first be performed. “Omics” approaches have been utilised to understand the state of the ECM in disease and tune implanted biomaterials accordingly. Proteome analysis refers to the systematic identification and quantification of the complete proteome of a biological system. For example, proteomics analyses suggest that compared with decellularised human lung, decellularised rat liver has different proteoglycan, ECM glycoprotein, and secreted factors content.^118^ These distinct molecular compositions could make these biomaterials useful for different biological applications. Determination of the matrisome components, which support cell growth, differentiation, and original tissue architecture and microvascular network, is useful to produce biomaterial with improved bioactivity. This analysis is also useful to determine compositional changes that occur in the ECM in disease processes.^119^ Quantitative proteomic analysis of ECM featuring characterisation of site-specific post-translational modifications on collagen reveals dynamic growth factor-mediated regulation of prolyl-3-hydroxylation and lysyl glycosylation.^120^ Characterisation of PTMs on proteins are essential to determine protein function and modified PTMs that are often implicated in disease.^121^ A database of PTM-disease associations, has been developed with detailed annotations to analyse further the relations between PTMs and their effect on protein function in human diseases.^122^ Proteomic analysis of the ECM has been coupled with decellularisation processing to study the native ECM structure in health and disease.^123^ In contrast, lipidomic profiling is performed less extensively. All tissue present specific lipid distributions and concentrations.^124^ Alterations of lipid homeostasis have been linked to cardiovascular disease,^125^ cancers^126^ and neurodegenerative diseases,^127^ among others.

Glycomic characterisation of tissues in physiological and disease conditions highlights the aberrant expression of glycans to identify markers of disease and inform the material design to address aberration.^128,129^ Recent technical advances have been made in glycomic analyses of *N*-linked and *O*-linked glycans, as well as glycosylated PTMs on the protein backbone of glycoproteins and proteoglycans.^130^ *N*-glycan retrieval by peptide *N*-glycosidase F, and subsequent isolation and characterisation protocols are well described, whereas *O*-glycans cannot be cleaved with an analogous enzyme as no such enzyme has been found in nature. Therefore, the *N*-glycome of cells and tissues is routinely analysed over other classes of glycoconjugates.^131^ *N*-glycome substrate and starting material characterisation will inform the bioactivity and specific material–host interactions.

Initial characterisation of a material using the techniques mentioned above offers insight into the molecular composition of the material, the natural modifications to material constituents and the innate bioactivity of such material based on the sequences and motifs present with known biological effect. Such a methodology may also be employed to assess the target tissue to match the molecular signature of a material to the intended tissue to provide the correct biological cues for implantation. Ultimately, the properties of all materials will be dictated by isolation methods used to produce starting materials and the source of the material. While the manufacturing techniques used to isolate polymers such as collagen for commercial use are beyond the review’s scope, it is essential to recognise that materials from different suppliers can vary greatly despite similar origins and batch-to-batch variability often cited.^132^ This necessitates careful consideration when comparing materials and results from different formulations, mitigated by in-house characterisation of starting materials.

## Material processing, cross-linking, and assembly

It is clear that natural polymers have innate biological properties that greatly influence cell and tissue responses; however, the processing and functionalisation of these materials can hinder final properties and potency. In this section, we consider processing techniques for the maintenance of bioactivity during the production of biomaterial-based devices. As mentioned previously, the use of natural-based polymers has increased exponentially due to their biocompatibility, biodegradability, and non-toxic properties.^133^ They provide not only necessary mechanical support due to their physical properties, but also bioactive traits that influence cell behaviour and tissue homeostasis, making them suitable candidates for regenerative therapies. ECM components, such as collagen, HA and proteoglycans, create porous network matrices that facilitate cell–matrix and cell–cell interactions. Therefore, when creating a biomaterial, it is necessary to consider the native tissue architecture and mimic this three-dimensional microenvironment through cross-linking, decellularisation, and purification techniques.

Organised networks are formed from materials such as collagen or fibronectin,^134^ which possess self-assembling properties, or starting materials coupled with cross-linking methods or a combination of both. Self-assembling properties of macromolecules may be lost during processing, depending on extraction, purification, and sterilisation methods necessary for their final application.^135,136^ Cross-linking modifications aim to fulfil the mechanical demands required by the application while also modulating the intrinsic biological properties of the natural polymer for the desired effect. Cross-linking methods can also be used in self-assembling polymers to modulate specific properties we wish to enhance or control, such as mechanical stability, degradation rate, and immunogenicity of the terminal device that will be implanted in the damaged tissue.^137^

Physical and chemical cross-linking are the two main strategies employed to form these polymeric networks, although some enzymatic approaches have also been explored.^138,139^ The site-specificity of cross-linking will determine access to biologically active motifs and function, potentially mask sites of immunogenicity and provide structural support.^140–142^ Cross-linking materials can be tuned to form enzyme cleavable linkages to promote remodelling and complete integration. However, natural elements can be considered to make materials resistant to degradation, such as glycosylated barriers.^143^ There is an abundance of tuneable features to be modulated when developing a scaffold to procure a suitable microenvironment for a given application. In this section, these cross-linking methods will be assessed, analysing the specific properties they provide to material structure and exploring the biological influences they may have on the material’s bioactivity (summarized in Table 3).

**Table 3:** Methods for biomaterial processing and considerations for retention of biologically active motifs

Cross-linking method	Reagents	Substrate	Cross-linking target	Preserved/modification of biological activity	Ref.
*Chemical cross-linking*					
Carbodiimide reaction	EDC/NHS DDC DMTMM DSG	Collagen, HA, elastin, gelatin, GAGs, etc.	Conjugates carboxylate groups present in integrin-binding motifs, such as RGD or CxOGER	May affect cell–matrix interaction. It is mitigated by a reaction occurring before the addition of peptide sequence	^140,195,196^
Aldehyde-reactive cross-linkers	Glutaraldehyde (GTA) Formaldehyde Genipin	Peptides/ polysaccharides (Collagen, gelatin, chitosan)	Amine groups in RGD sequence	Charge-shift modulates cell-fibre interaction	^137,206,207,337,338^
Amine-based reaction	NHS ester DMA DMP DMS	Collagen, Chitosan	Amine groups in N-terminal of peptides and polymers/ side-chain of lysine amino acid residue	Increase of amine availability by modifying the pH during gel’s formation. Alters motif integrity and function, for cell-adhesion or proliferation	^141,211,339^
Thiol-based reaction	Maleimides Haloacetyls	Alginate, thiol-modified chitosan, gelatin, HA, etc.	Sulfhydryl groups of cysteine amino acids. The secondary or tertiary structure of proteins	Toxic by-products induce cell apoptosis, off-target thiol cross-linking inhibits protein function, masking of cleavable sites for remodelling	^203,204,340–344^
Photo-based cross-linking	Diazirine Aryl azides	Gelatin Collagen, HA, fibrin	Amine groups in the N-terminals of peptides and polymers and the side-chain of lysine amino acid residue	Blocks MMP accessibility to RGD sequence, for degradation	^225,226,251–253,341,345,346^

Cross-linking method	Examples	Mechanism	Preserved/modification of biological activity	Tuneability	Refs.
*Physical cross-linking*					
Ionic/electrostatic	Chitosan Alginate Chondroitin sulphate	Opposite charges between polymer sequences	The charge density of polymer creates a highly porous network	Allows nutrient exchange to support cell encapsulation and drug delivery	^183,186–188,192,254,255^
Hydrophobic interaction (Thermal induction)	Methylcellulose Collagen Elastin-like peptides	Hydrophobic interaction induces self-assembling	Formation of micelles due to temperature stimulus that results in situ gel cross-linking	Cell differentiation, carry biologically active molecules, or enhance cell adhesion	^166–168,173^
Hydrophobic interaction (Ultrasonic induction)	Silk fibroin Hyaluronic acid Collagen	Proteins beta-sheets self-assemble in the crystalline structure	Macromolecular interactions. Changes in collagen molecule structure and final organization, promoting cell migration	Decrease cell attachment to the collagen fibres. Enhancement of cell migration allows ECM remodelling	^174,175,347^
Crystallisation	Sodium alginate (SA)	Crystal formation from freeze-thawing cycles	Increased conductivity after gel’s formation	Promote cell differentiation and proliferation, inflammatory phenotypes	^348–351^
Host-guest interactions	Cyclodextrins Crown Ethers Cyclophanes Cryptophanes Porphyrins	Host molecule void is replaced by complementary guest molecule	Development of self-assembled membrane-like structures that mimic glycocalyx	Allow cell recognition and adhesion through lectin-receptors	^352^

Processing method	Starting material example	Process description	Final product	Advantages/retained activity	Refs.
*Decellularisation and processing techniques*					
Lyophilisation/ Pulverisation	Notochordal cell matrix (Tissue)	The tissue is lyophilised, resulting in a dry and brittle matrix, and subsequently pulverised using a microdismembrator	Viscous fluid made up of proteins, proteoglycans and soluble factors	Retained biologically active proteins, e.g., preserved anabolic and anti-inflammatory effects, as well as lubricating properties	^353^
Decellularisation	Porcine-derived ECM	Mechanical delamination	Organ derived ECM-based biomaterial	Preserved native ECM components and structure mediates cell/tissue—biomaterial interactions such as fibronectin-integrin binding via RGD and REDV motifs	^354^
	MEF-derived ECM	Chemical decellularisation	ECM-based biomaterial derived from cell culture	Promoted cellular responses such as proliferation and differentiation due to preserved properties of the biomaterial	^355^
	Tendon-derived ECM	Mechanical and chemical decellularisation combined	Tendon derived ECM materials	Activation of signalling pathways and cellular proliferation/differentiation via retained integrin molecules within the biomaterial	^356^
α-Gal epitope removal	Bovine, porcine heart valves	Tissues were treated with the α-galactosidase enzyme	Removal of α-Gal epitopes from the cell surface of the tissues	α-Gal epitopes play a role in calcification and degradation of valves and result in implant rejection. This process modulate the immune reaction to biomaterial	^357^

*DDC* dicyclohexylcarbodiimide, *DMA* dimethyl adipimidate, *DMP* dimethyl pimelimidate, *DMS* dimethyl suberimidate, *DMTMM* 4-(4,6-Dimethoxy-1,3,5-triazin-2-yl)-4-methylmorpholinium chloride, *DSG* disuccinimidyl glutarate, *EDC* 1-ethyl-3-(3-dimethylaminopropyl)carbodiimide hydrochloride, *GTA* glutaraldehyde, *GxOGER* domain of glycine-x-alanine–hydroxyproline–glycine–glutamate–arginine, *MW* molecular weight, *NHS* N-hydroxysuccinimide, *RGD* tripeptide domain of Arg-Gly-Asp

Higher concentrations of cross-linker involve a higher number of covalent bonds in the polymer network, and as a result, there is an increase in the stiffness and a decrease in the degradation rate of the biomaterial.^144,145^ These changes can modify protein structure and drop the affinity in binding motifs, may ultimately affect the biological function of a protein or drop their enzymatic activity.^146^ Also, increasing the amount of crosslinker, and thus the number of the chemical agent’s functional groups, is linked to higher risks of cytotoxicity.^147^

Several aspects must be considered when designing a biomaterial that will be chemically cross-linked. To begin with, what family of cross-linking agents will be used according to the functional groups that will be involved in the covalent bond (NHS ester reaction or maleimide reaction, for instance). Other important aspects that should be considered are the functionality, cleavability, structural modifications, spacer arm composition, length and structure of the conjugating agent, etc.

Degradation and subsequent by-products must be considered designing a biomaterial set for regenerative therapies. Matrix digestion by metalloproteases, allows cells to recognise ECM degradation products to synthesise and reorganise a new matrix.^148^ While it is essential to minimise the degradation products of synthetic materials, natural polymers also degrade to produce deleterious by-products with undesirable biological effects. This effect is exemplified by hyaluronic acid where high molecular weight HA molecules exert an anti-angiogenic effect, highly present in avascular tissues such as cartilage. However, under high degradation rates, producing smaller HA products, are associated with neovascularisation and pro-inflammatory response, thus playing an essential role in wound healing, but also tumour progression and metastasis.^149^ This effect may be mitigated through an optimised degradation profile and observed cellular response.

Several methods of cross-linking are discussed below; however, it is important to note that these methods are almost entirely limited by the initial starting material unless further modified. Thus, we have considered the additional biologically active characteristics encompassed by these techniques to offer further insight into method suitability for given applications.

### Physical cross-linking

Physical cross-linking may be divided into ionic/electrostatic interactions and hydrophobic/hydrophilic interactions. Physical cross-linking is based on the formation of reversible intermolecular interactions. This approach’s main advantage is the low cytotoxicity, assured by the absence of chemical agents involved in the reaction. Cross-linking is triggered by certain stimuli, such as pH or temperature. At the same time, the physical attraction that forms the crosslinked product can be disrupted at high stresses but restored once the force is removed, providing these materials with self-healing capacities and injectable properties under room temperature.^150,151^ However, the mechanical loading forces that these materials can sustain is limited due to the reversible binding of physical interactions in this network.^152^ Self-assembling polymers also demonstrate physical cross-linking through various interactions and are thus discussed separately.

Ionic/electrostatic interactions are based on the reversible bonding between two opposite charges that come from either ions or macromolecules formed by positively or negatively charged groups.^150^ These facilitate the formation of polyelectrolytes complexes (PECs) to generate a network.^153,154^ For example, chitosan is a positively charged polysaccharide, formed by *N*-acetylglucosamine and glucosamine residues. It can form polyelectrolyte complexes (PECs) via electrostatic interactions between its cationic groups and the anionic groups from various anionic macromolecules such as pectin,^155^ chondroitin sulphate,^156^ and alginate.^157^ Alginate, a polysaccharide composed of mannuronic and glucuronic acid residues, provides anionic charges to its structure and allows crosslinking with divalent cations such as calcium, barium, or magnesium, among others. These divalent ions will serve as reactive bridges to other anionic molecules or more alginate chains, resulting in a gel structure.^158^ Alginate beads show high mucoadhesive properties, prolonging their residence in mucosal tissues showing great potential for mucosal drug delivery.^159^ Also, the porosity of ionic alginate hydrogels allows nutrient exchange, making them perfect for cell encapsulation.^159,160^ The viscoelasticity shown by ionic crosslinked gels better mimics the natural properties of tissues and can influence cell behaviour.^161,162^ However, the release of cross-linking ions due to the disintegration of the alginate beads can lead to homeostatic imbalances in resident cells, affecting the response of the tissue to the therapeutic effect of the material or encapsulated drugs.^163^ Mammalian cells lack degradative enzymes to cleave alginate polysaccharide chains and thus, remodelling is inhibited. Interestingly, the mechanism for physical cross-linking that involves chelation of Ca^2+^ is a potent antibacterial motif with the capability of disrupting biofilm.^164^ This chelating site has also been shown to bind other monovalent and divalent ions, to attenuate hypertension through increased salt excretion when administered systemically in a rat model.^165^ These ionic properties attributed to functional groups can be further tuned by chemical substitution at the polysaccharide chain: ionic group substitution effects cell–matrix interactions, growth factor binding and cell migration and proliferation.

Hydrophobic interactions involve water-soluble polymers with hydrophobic end groups, side chains or monomers, where polymerisation occurs due to thermal or ultrasonic induction by amphiphilic blocks that contain both hydrophobic and hydrophilic groups that make them suitable for self-assembly at a given temperature. These properties enable soluble formulations at room temperature, to form gel-solid structure under physiological conditions at 37 °C.^166^ Methylcellulose can form thermoresponsive hydrogels as well as gelatin^167^ or elastin-like polypeptides (ELPs), among others. ELPs derive from the recurring amino acid sequence Val-Pro-Gly-Xaa-Gly (being Xaa any amino acid except Pro), that can be found in the hydrophobic domain of tropoelastin.^168^ They can be used in many biomedical applications, including differentiation stem cells,^169^ drug delivery^170–172^ or formation of layer systems with other polymers that can ultimately be used as coatings to enhance cell adhesion or carry biologically active molecules.^173^ The highly repetitive sequence GAGAGS of some natural proteins such as silk fibroin, allow the formation of the antiparallel beta-sheet crystalline region that permits its self-assembly into an organised structure through methods such as ultrasonic induction.^174^ Cross-linking can also be used to modify specific biological properties of the polymer, where the degree of ultrasonic modifications alter the triple helical structure of collagen to modulate integrin-mediated cell attachment and facilitate cell migration and ECM remodelling.^175^ Hydrophobic interactions do not exclusively involve material interactions, but also encompass cell–material interactions, heavily influencing non-receptor mediated cell adhesion, protein adsorption, and activity of surface functional groups.^176^ Further discussions on these interactions is beyond the scope of this review, however, these interactions are highly cell and material dependent.

### Self-assembly

Some of the natural polymers used for biomaterial design have self-assembling properties, meaning they naturally generate non-covalent intermolecular interactions between them to form these networks. Most of the interactions involved in self-assembly involve non-covalent interactions, such as hydrogen bonding, Van der Waals forces, electrostatic forces, hydrophobic interactions or π–π stacking of aromatic rings, like those involved in physical cross-linking.^177^ These non-covalent interactions promote the assembly of many native polymers and structures into a higher level of structural complexity, such as lipids into micelles or bilayers and α-helix or β-sheet structural motifs of proteins.

Some examples of self-assembling macromolecules include DNA,^178^ proteins and peptides,^179^ lipids,^180^ etc. These elements possess certain structural features that allow their self-polymerisation. Collagen most notably self-assembles with fibrillar conformation through alpha helical formations.^181^ Laminin and elastin possess similar properties while fibronectin’s N-terminal domain (especially the domain I1-5) allows the assembly of its dimers in a 3D-structure.^179^ Ternary complexes are formed by cooperative interactions between repeats I_1-5_, III_1_ and the cell or heparin domains, which are thought to be important during fibrillogenesis.^182^

Lipids such as fatty acids self-assemble due to hydrophobic interactions, assembling and forming bilayer structures when exposed to water. This characteristic makes them suitable in bioengineering as delivery systems of molecules or drugs.^180^ Hydrogen bonds and dipolar interactions between lipid monomers can derive to stiffer gels or form lipid rafts, which exert essential biological functions,^183^ such as signal transduction at the cell surface in hematopoietic cells.^184,185^ Phospholipid modifications can also be applied to peptides to allow self-assembly formation and enhance cellular internalisation. This is exemplified by adding palmitoyl tails to a cell-penetrating peptide to provide with hydrophobic capacities, and enhance self-assembling behaviour to develop an intracellular delivery system for hydrophobic drugs internalised into these bilayer carriers.^186^

The principal for self-assembly relies on complementary ionic bonds involving amino acid sequences alternating positively-charged and negatively-charged (Lys and Arg mainly used as positively-charged residues, whereas Glu and Asp are used as negatively-charged forces).^187,188^ This capacity is encoded within the primary amino acid sequence of the peptide, but the process usually requires the interaction with external molecules, for instance, integrin α_5_β_1_ cooperation to promote fibronectin’s assembly.^189^ This property can be tuned by modulating the concentration of these external molecules involved in the reaction, by adjusting the interactions between the molecules and the material or by adding an external stimulus into the formulation for instance, such as pH, concentration, ionic strength, or temperature,^190^ similar to the physical cross-linking interactions previously described. Considering these are non-covalent bonds, they are incredibly weak when considered individually, but can derive to highly stable assemblies when many of these interactions are combined. To preserve the natural self-assembling properties of starting materials, cross-linking methods or functionalisation through conjugation steps retain accessibility to essential motifs to facilitate interaction and assembly. This technique of structural organisation is advantageous for avoiding toxic by-products of chemical cross-linking reagents in sensitive cell and tissue types.

### Chemical cross-linking

While physical cross-linking is often a natural property of macromolecules, it may not suffice for the mechanical properties necessary in a given application. Chemical cross-linking is based on the bond between functional groups present in the primary structure of a polymer (such as carboxylic groups, hydroxyl groups, or primary amines) with the reactive groups given by a cross-linking agent (such as aldehydes, carbodiimides, etc.). As a result, a covalent bond is obtained, to enhance biomechanical stability and tuneability for degradation.^152^ Chemically crosslinked materials can achieve more excellent stability, more extended durability, and higher mechanical properties compared to physically cross-linked substrates. Biomaterials have been crosslinked with a vast range of chemical approaches, including photo-polymerisation, enzyme catalysed reactions, “click chemistry”, Schiff base formation, oxime crosslink, Michael addition of dynamic covalent chemistry.^150,152^ However, in this review, we focus on the reactive groups most commonly associated with biological motifs in biomaterial research and their implications and effects in the final function.

One of the most common cross-linking strategies is based on the carbodiimide chemistry. The reaction involves carboxylic groups, present on the starting material that will interact with the primary amine groups of peptides sequences,^191^ or a synthetic polymer functionalised with amine groups, to generate a stable covalent-based network. EDC (1-ethyl-3-(3-dimethylamino propyl-carbodiimide hydrochloride) is widely used together with NHS (N-hydroxy-succinimide), to react with the carboxylic groups and generate a reactive ester group that can bond to the primary amines.^192,193^ Nonetheless, despite being an efficient approach for the mechanical stabilisation of, for instance, collagen,^191,194^ EDC utilises most of the carboxylate groups of the primary structure provided by glutamate or aspartate. These amino acids are essential for the interaction with integrin-cell receptors in collagen and gelatin^195,196^ in the cell-binding motifs CxOGER and RGD (tripeptide domain of Arg-Gly-Asp), respectively. In this regard, many strategies have been explored to restore this functionality, such as cross-linking triple helical peptides (THP) to synthetic polymers in order to mimic the lost CxOGER motif and replicate the collagen’s cellular binding capacity.^197,198^ It can also be bound to the same collagen by conjugating the pre-designed THP sequence to a photoreactive moiety, diazirine, allowing ultraviolet light (UV)-dependent covalent coupling to collagen films.^199^ This approach has been reported to re-establish not only integrin-binding interactions,^197–199^ but also Discoidin Domain Receptor 2 (DDR2) and the A3 domain of VWF, both critical for wound healing and myocardial repair.^200^ Other strategies have also been explored for RGD motif restoration.^201^

Other chemical cross-linking methods widely used in the biomedical field are amine-based reactions, where the amine reactive groups in the surface structure of the polymer are being utilised for crosslinking. Examples of such crosslinkers include NHS ester, imido esters, pentafluorophenyl ester, and hydroxymethyl phosphine.^202^ An increase in the degree of cross-linking is translated into a reduction of the reactive groups available in the primary sequence, which may affect the motif integrity or influence its function. Thus, modulating the degree of cross-linking and thus, the number of free amines still available can have a significant impact in the biomaterial’s biological function. For example, in a chitosan/tripolyphosphate scaffold, the amine availability is determined by the pH of the solution and therefore influences the proliferation of bone marrow mesenchymal cells.^141^ This can be applied not only to amine cross-linking, but also to other reactive groups. Crucially, this amine-based reaction selectively attacks the *N*-terminus of peptides, preserving biologically active sites and cell–matrix interactions; however, this selectivity is dependent on reactive ester synthesis, which may non-specifically target sequences.

Another mechanism includes sulfhydryl reactive groups as a core for cross-linking. In contrast with the previous examples, this group tends to be part of the protein’s secondary or tertiary structure in the side chain of cysteine residues, which generate intermolecular interactions via disulphide bonds.^203,204^ Therefore, to make these groups available for others and crosslink, they must be reduced to sulfhydryl groups. Thiol-acrylate reactions are commonly referred to as “click reactions”, and they have been used to incorporate specific functional motifs to a cysteine-based cross-linker. The principle of this approach is to incorporate certain functionalities in a biomaterial to enhance cell attachment through integrin interaction, for instance. This strategy is mainly conducted through thiols due to their high reactivity, and also they are easily quantifiable to keep track of the cross-linking units used.^205^ This method of chemical cross-linking is specific for cysteine containing sequences, and preserves remaining biologically active sequences for bioactivity.

Aldehyde reactive groups can also be applied for cross-linking, by oxidation of carbonyl groups in carbohydrates to generate covalent bonds.^137^ Examples include hydrazides or alkoxyamines, while glutaraldehyde (GTA) is one of the crosslinkers mostly used, reacting with primary amines of the polymer, such as collagen.^206,207^ Usually, it reacts only with the surface of the molecule, leading to intermolecular crosslinks that allow the bonding between collagen fibres and leading to very stable networks. However, GTA shows high cytotoxicity effects^208^ and alternative crosslinkers can be used to provide similar enzymatic stability but to lower cell mortality, such as genipin.^207^ Beside of its cytotoxicity, it has been shown that GTA cross-linking can induce a charge shift in the collagen fibre, reducing the positive charges without disrupting the overall structure of the macromolecule, having thus implications on the behaviour on cell response and attachment to the scaffold.^209^

Photoreactive crosslinkers have also gained much attention, especially for in situ gelation approaches.^137,210^ An example of photochromophore includes, for instance, diazirine.^199,200,211^ Among their multiple applications, such as drug delivery, photoresponsive hydrogels have also been used as a matrix for cell cultures. These photomotifs can promote oligosaccharide release in hydrogel matrices, or promote the oligomerisation of hydrogel matrices containing binding motifs such as RGD, which in turn interacts with integrin-complexes in cell membranes, being able to guide cell growth and differentiation.^212–214^ Moreover, the degradation of the hydrogel can also be modulated via light-stimulus, being able to regulate cell migration of stem cells, making photo-polymerisation an attractive cross-linking approach.^215^

There is a multitude of cross-linking techniques available in tissue engineering, each with variable specificity, toxicity, degradability, and mechanical properties. While cross-linking approach may be dictated by the mode of delivery for a biomedical application, it is worth-considering the sequence specificity of cross-linking initiators and the subsequent impact on bioactive motifs of the starting polymer.

The use of cross-linking methods with one specific substrate can mitigate off-target reactions that interfere with antigen and motif expression. However, this becomes exponentially more complex if the material substrate is a mixture of proteins, GAGs, lipids, etc. In this case, an interpenetrating network consisting of an artificial substrate to create a synthesised matrix that contains natural components may be favourable depending on the target.

## Isolation and processing to preserve the natural architecture

While using macromolecules as starting molecules, further crosslinked to form mature networks is an attractive approach to material design, decellularisation techniques for organs and tissues have obvious structural and biological advantages. The basic principle of the decellularisation process is removing the cellular content of ECM while preserving its native microstructure, molecular composition, and surface chemistry.^216^ Decellularised substrates contain more significant structural organisation with specific biological cues over crosslinked substrates. A key challenge to the clinical translation of decellularised scaffolds lies in processing techniques to remove cells and immunogenic motifs from the starting material, while maintaining the bioactive properties and ECM architecture to promote scaffold integration, cell proliferation, and tissue regeneration.

Current standards for evaluating decellularised products focus on remaining nucleic acid content and removal of known immunogenic epitope such as alpha-galactose using enzymatic treatments.^217^ Although the no authority has strictly defined standards for decellularisation, criteria cited includes no visible nuclei, DNA content should not exceed 200 base pair length and total DNA content should not exceed 50 ng per mg of material.^218^ However, these techniques fail to capture numerous proteins and polysaccharides that induce immunogenic responses.

Design of biomaterials from natural polymers aims to optimise the substrate’s inherent bioactivity to promote tissue regeneration by identifying and preserving bioactive components to ensure high potency final product. From starting material isolation to final processing, material treatments to remove antigenicity and contaminants must not perturb the natural structure beyond biological activity. Non-mammalian polysaccharides are unlikely to induce TLR receptor activation, but rather impurities in an isolated starting material are responsible for an inflammatory response. While TLR ligands include carbohydrate moieties, the non-carbohydrate portion of molecules such as lipopolysaccharide activates receptor signalling pathways.^219^ The effect of TLR’s carbohydrate-binding domains is unknown, although it has been shown that inhibition of polysaccharide binding inhibits macrophage activation.^220^ Purification techniques must be optimised to remove pro-inflammatory antigens while retaining desired material properties.^216^

Decellularisation efficacy and complete removal of cellular components are essential for the biomaterial compatibility and biomaterial–host interactions.^221–223^ Physical processes and sonication, chemical detergents, and enzymatic treatments have been used in combined decellularisation procedures.^117^ However, these decellularisation techniques can alter the structural integrity and native function of ECM components.^224,225^

Physical degradation includes snap freezing and lyophilisation, freeze-thaw cycles and mechanical destruction. Physical methods are useful as they do not perfuse the tissues with harmful solvent or chemicals yet these techniques disrupt ECM organisation, which is crucial for applications such as cardiac valves and ligaments.^226^ Solvent extraction (ethanol) is useful for membrane removal; however, prolonged exposure can disrupt protein integrity.^227^ Acidic treatments are used for decalcification as well as decellularisation but these cause oxidative damage to GAGs and collagen.^228^

Detergents can be classified as non-ionic (Triton X-100), ionic (sodium dodecyl sulphate), cationic (Cetyltrimethylammonium bromide, CTAB) and zwitterionic (3-[(3-cholamidopropyl)dimethylammonio]-1-propanesulfonate, CHAPS). These are used to disrupt lipid membranes, induce cell lysis and denature proteins. While non-ionic detergents preserve protein–protein interactions, ionic detergents tend to denature proteins.^229^ Also, cationic detergents induce structural changes in ECM components while zwitterionic compounds generally induce minimal ECM disruption although these are often used in conjunction with endonucleases to ensure DNA disruption.^230^ Detergent removal and protein purification must also be considered using dialysis, gel filtration, and column separation depending on detergent used and end application.

Enzymatic treatments degrade cell proteins and antigens and reduce DNA content in decellularisation protocols. Trypsin is a serine protease commonly used as an enzymatic decellularisation that effectively cleaves peptides of unwanted proteins. However, it will disrupt ECM components with prolonged incubation times and is entirely unsuitable if the bioactive molecule to be retained is a K/R containing peptide.^231^ Endonucleases and exonuclease cleave phosphodiester bonds between nucleotides to remove immunogenic nucleic acids, yet these enzymes themselves are immunogenic and can be difficult to remove from the final product. Decellularisation protocols vary based on the source and characteristics of the tissue.^216^ Optimisation of processing techniques are required to preserve the native properties/motifs of the ECM (or macromolecules within the ECM) depending on whether the active component of the material is protein, carbohydrate, or lipid-based.^225,232^ Such optimisation is further complicated by complex matrix products with multiple active components. In these mixtures, there must be a trade-off between physical methods of decellularisation, which do not retain tissue architecture, and detergent methods with potential motif denaturation.

## Final characterisation to investigate retained activity

Optimisation of biomaterials is often assessed by the final characteristics of a range of materials to dictate the final optimised formulation. While products must satisfy mechanical testing and biocompatibility studies to be considered, increased focus has been targeted towards developing bioassays to mimic tissue and disease microenvironments to assess the product’s biological effect. This assessment of materials’ efficacy and potency may be a useful screening tool to rule out potential candidate formulations, saving costs on ex vivo and in vivo studies down the line. Herein, we discuss several methods of investigation to determine the biological activity of biomaterial products (Table  4).

**Table 4:** Characterization of retained bioactivity of natural molecules and substrates

Assay	Substrate	Description	Outputs	Relevance	Refs.
Cell-biomaterial interaction	Biomaterial product	Biosensor based on an array of 20 μm fibronectin circular isles to facilitate the performance of cell adhesion assays and posterior affinity analyses	Evaluation of the cell adhesion kinetics, the integrin profiling and their contribution to cell attachment and adhesion	Integrin profiling gives information regarding to cellular adherence, cell–biomaterial interactions and mechanosensing properties of biomaterials	^240^
2D/3D scaffold variability	Biomaterial product	2D systems for integrin-specific cell binding on surfaces and protein films coated with collagen, gelatin and collagen/gelatin compositions. 3D scaffold discs (collagen based) evaluate non-integrin specific morphological features	Characterised binding motifs	Assessment of adhesive properties and cellular attachment for downstream signalling pathways, to influence cell and tissue fate	^70^
Mechanical assessment	Cellularised product	Contractility of the material is measured by dynamic mechanical analyser, to determine the effect of cells on material mechanics	Altered biomechanics	Confirmation of binding and cytoskeletal activation. Assessment of mechanical properties of natural material after processing	^358,359^
Capillary electrophoresis	Biomaterial product	Capillary affinity electrophoresis was performed on Beckman MDQ glycoprotein systems platform/ Ar-laser-induced fluorescence detection system	Characterised interactions between HA oligosaccharides and hyaluronan-binding proteins (HABP)	Identified interactions and structures can be confirmed based on theorised structure	^233^
ECM microarray platform	Collagen type I, Collagen type III, Collagen type IV, laminin and fibronectin	Mixture of ECM macromolecules in different proportions were placed on hydrogel slides by using a standard DNA microarray to assess cell–biomaterial interactions	Pre-mixed blends of biomaterials to allow the rapid identification of cell–material interactions	This platform enables the study of cellular responses to a multitude of ECM biomaterials in parallel. It provides rapid bioactivity assessment for biomaterial with desired functionality	^360^
Single-cell transcriptome analyses	Composite hydrogel of alginate and matrigel	RNA sequencing was performed on 2D/3D cell cultures treated with matrigel and biomaterial composites, by using Smart-seq2 protocol. Transcriptome analyses were run on RNA-Seq by expectation maximization (RSEM) and Cell ranger platforms	Effect of cross-linking degree on transcriptomic profile of cells	Cell-biomaterial interactions, biomaterial induced biological processes, effect of biomaterial processing on these can be identified	^259^
Proteomics Analyses	Organ-derived ECM biomaterials, Collagen type I gel and GFR-Matrigel	LC-MS/MS tandem mass spectrometry were performed on HPLC coupled to an ESI ion-trap/Orbitrap mass spectrometer platform. Proteomic data were analysed by MaxQuant programme	ECM glycoproteins and regulators, proteoglycans, secreted factors	Assessment of molecular composition of ECM derived materials is vital to understand material structure and activity for tissue engineering application	^118^
IHC staining	Decellularised porcine aortic valves	IHC staining was performed for elastin, collagen, proteoglycan, GAGs, fibronectin and laminin	Effect of decellularisation processes on ECM molecular content was assessed	Effects of material processing can be evaluated, and protocol optimization can be done based on the IHC staining	^361^
Mechanical Assessment	Decellularised organ derived biomaterial	The mechanical response was examined by uniaxial ring test on biomechanical tester platform, before and after the decellularisation process	Mechanical properties of native tissue were preserved	Preservation of mechanical properties of biomaterial, such as stiffness and elastic modulus, is crucial for tissue response since cells can provide response to mechanical properties/mechanosensing	^362^
Lipidomic analysis and RNA sequencing	ECM-based biomaterial	Lipidomics analyses were performed with a Dionex Ultimate 3000 HPLC system coupled online to a Q Exactive hybrid quadrupole- orbitrap mass spectrometer. RNA sequencing was performed on NextSeq instrument	Catalogued differentially expressed miRNA, phospholipid and LPL profile, free and oxygenated fatty acids	Identification of miRNA profile involved in cellular growth, development, proliferation and morphology. Phospholipids involved in signalling pathways crucial for cellular fate. Lipidomics is an essential tool for tissue repair and remodelling	^258^

Initial tissue and substrate characterisation informs material design and optimization to produce a device with high fidelity to natural tissue modifications and biological activity

While initial tissue characterization techniques aim to identify crucial and unique biological motifs through multiomic studies, the same techniques can evaluate biomaterial products that aim to replicate the same tissues. In addition, screening methods may be used to investigate post-translational modification of proteins with carbohydrates using capillary electrophoresis.^233^ Such methods allow classifying a complex mixture of carbohydrate chains to examine the binding between HA oligosaccharides and hyaluronan-binding proteins (HABP), for instance.^233^

3D scaffolds can be designed to increase cell adhesion through optimisation of construct architecture, physical parameters, and chemistry (available binding sites). Evaluation of these materials requires the rapid and reliable assessment of biological motif availability and subsequent integrin recognition, which may be compromised during processing.^70^ 2D systems may provide a reliable way of screening a broad range of compositions and treatments such as cross-linking on integrin-specific cell binding. At the same time, 3D scaffolds reveal morphological features responsible for a significant amount of non-integrin specific interactions on scaffolds, which should be considered when assessing the biological activity of 3D substrates. Systematic alteration of material properties can be screened using these assays to optimise biological activity.^70^ While much focus has been on modifying these naturally occurring macromolecules to optimise given properties, further characterisation, and utilisation of intrinsic properties are needed to enhance tuning of these materials to fulfil the desired function.

ECM properties and signalling cues are tightly regulated to ensure normal development, remodelling, and physiology.^234^ Homeostasis is an active process that must be continuously maintained and not a passive state. Therefore, maintenance requires precise processing in a biological system, through matrix sensors and consequent effector mechanisms to achieve continuous equilibrium. This fact must be emphasised in designing implantable materials to promote reciprocal crosstalk between cell-sensing components and the surrounding microenvironment. Even robust materials will fatigue over time and require replacement. Materials must promote cell integration and, more importantly, scaffold turnover for complete integration and sustained maintenance over time. Even at steady state, tissues and organs have to maintain structural integrity and functionality dynamically.^235,236^ The potential of naturally-derived scaffolds arises from the present biochemical and biophysical cues, that preserve ECM homeostasis and mechanical properties mostly by influencing resident cells.^237^

**Fig. 3:**
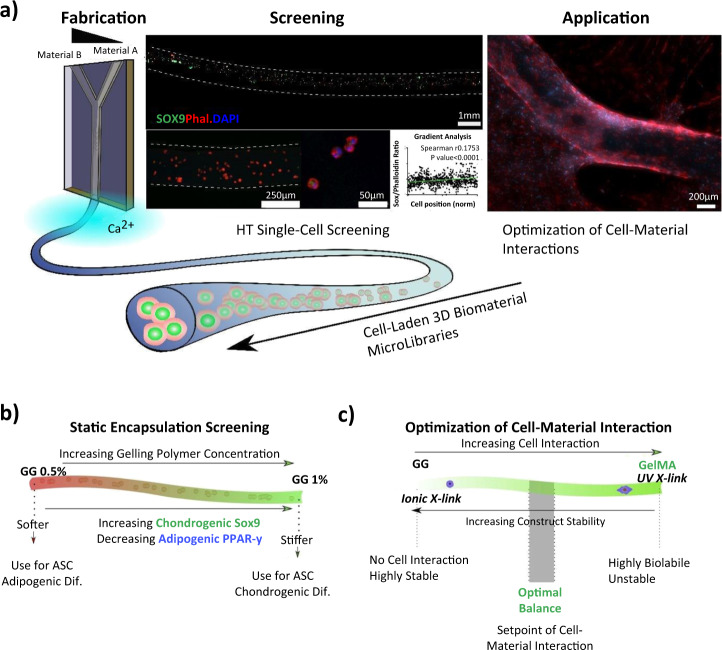
A fully high-throughput (HT) microfluidic platform for the generation of novel multi-material, multi cross-linking 3D cell-laden gradients as screening libraries (**a**). This high-throughput can be used to fabricate gradient libraries of polymer concentration to investigate the variable resultant cell phenotype to optimise material properties (**b**), or to optimise the cross-linking of materials and method of cross-linking to investigate cell–matrix interactions (**c**). Such a gradient platform allows for rapid screening of adhesion-related responses to determine material formulations for optimal function. In this study, cell-interactions were promoted with increasing UV cross-linking. Reproduced by permission of The Royal Society of Chemistry- Mater. Horizons (2020)^363^

Adhesive properties of biomaterials enable them to interact with the cells in the tissue microenvironment.^238^ Cellular adhesion to biomaterial activates certain cellular signalling pathways and promotes cellular proliferation, differentiation, and migration.^239^ Bioactivity of the biomaterial can be assessed through microarray based biosensor methods.^240^ These microarray based methods give insight about cell adhesion kinetics, integrin profiling, and non-specific interactions. This platform enables to perform adhesion profiling of various ECM components with multiple cell types in parallel. Further, ECM–cell interaction analysis is exemplified by the fully high-throughput (HT) microfluidic platform described in Fig. 3. This assay enables the generation of novel multimaterial, multicross-linking 3D cell-laden gradients as screening libraries to establish cell–material interactions and assess the optimal material formulation based on cell responses.

In order to achieve a successful tissue regeneration and restoration, biomaterial scaffolds must mimic the mechanical and porous structural network of the ECM, hierarchical architecture and surface chemistry, which enable cell–cell, cell–ECM interactions, and cellular behaviours. Preservation of material secondary/tertiary structures is essential for scaffolds to perform their native biological activities. Characterisation techniques such as scanning electron microscopy, transmission electron microscopy, and atomic force microscopy have been used for morphological analyses of biomaterials.^241–245^

Identification of the molecular content of ECM-derived or decellularised materials plays an essential role in the assessment of tissue–material interactions and biocompatibility. The molecular composition of ECM-derived biomaterials depends on the tissue/organ source and processing techniques.^223^ For example, proteomics analyses suggest that compared with decellularized human lung, decellularized rat liver has different proteoglycan, ECM glycoprotein and secreted factors content.^118^ These distinct molecular compositions could make these biomaterials useful for different biological applications. Determination of the matrisome components, which support cell growth, differentiation, and original tissue architecture and microvascular network, is useful to produce biomaterial with improved bioactivity. Additionally, proteomics tools help the identification of structural and molecular loss during material processing.^118,246–250^ Preservation of vital ECM components such as collagen, laminin, fibronectin, and other ECM regulators affects the biological response of the tissue and success of the transplantation.^251–253^

Determination of phospholipid composition of ECM-derived biomaterials is crucial since they can act as a precursor of signalling molecules and involve in cellular processes and responses.^254,255^ Lysophospholipids (LPLs) involve in immune cell activation, tissue inflammation and fibrosis formation, and tissue regeneration.^256,257^ Liquid chromatography-mass spectrometry (LC-MS) based lipidomic and redox lipidomic tools can be utilised to identify tissue response and biological activity of the biomaterial.^258^ Effects of lipidome profile of ECM-derived biomaterials are unknown and needed to be further investigated. Transcriptomic analyses can be performed in order to understand cell–biomaterial interactions. Biomaterial composition, architecture and mechanical properties affect the biological responses in the tissue. Effect of biomaterial processing and design on biological processes can be identified on a single cell transcriptomic level.^259,260^

## Applications of material design using bioactive properties of natural polymers

Thus far, we have presented a comprehensive review of natural molecules and polymers used in tissue engineering, given an overview of their intrinsic biological properties and discussed cross-linking and processing techniques to retain biological activity, validated by bioassays and final assessments. Further, we present several examples of devices and systems that have employed a similar workflow based on natural material properties, incorporating initial material characterisation and final assessment of natural properties to optimise device design, streamline processing and enhance product potency and efficacy. Cell carrier systems are the leading example of natural polymer systems utilisation to take advantage of intrinsic bioactivity and enhance the tissue–biomaterial interaction, thus improve the clinical response.^261^ Delivery of the cells restores damaged tissue structure, components, function. In contrast, the carrier system enhances cell survival, proliferation, and protects cells from damage through delivery. Both implantable and injectable biomaterials have been used as carrier systems.^262,263^ Maintaining the desirable cellular phenotype and differentiation, especially in stem cell delivery, is crucial for a satisfactory clinical output. Cell–matrix, cell–biomaterial, and biomaterial–tissue interaction after the transplantation mediates ECM remodelling.^264^ Natural macromolecules such as collagen, hyaluronan, and gelatin have been used as cell delivery matrices due to their ability to promote desired cell function and phenotypes with their native properties.^71,265,266^

HA has been widely investigated for its anti-inflammatory properties and swelling capabilities when formulated as a hydrogel. The anti-inflammatory effect of high molecular weight hyaluronan (HMW HA) through its interaction with IL1-β receptors, nerve growth factor, brain derived neurotrophic factor, and interferon α2β (IFNα2β) signalling pathway.^267,268^ HMW HA microgels showed significant anti-inflammatory properties by downregulating the IFNα expression in an in vivo tail disc injury model. Hyaluronan can interact with the cells through binding with the CD44 receptor.^269^ This property of HA in cellular delivery has been utilised in our laboratory. Increased CD44 expression in Nucleus Pulposus (NP) cells showed that cross-linked HA hydrogels prevent further inflammation by binding CD44 receptors on NP cells, thus inhibiting the pro-inflammatory cytokine binding to this receptor.^268^ The survival and integration of retinal stem cell-derived rods in the retina have been mediated with the activation of survival pathways through HA biomaterial–CD44 receptor interaction between retinal stem cell-derived rod cells.^265^ The efficacy of CD44 targeting is reflected by the number of hyaluronic acid based systems reaching clinical trials.^270^

**Fig. 4:**
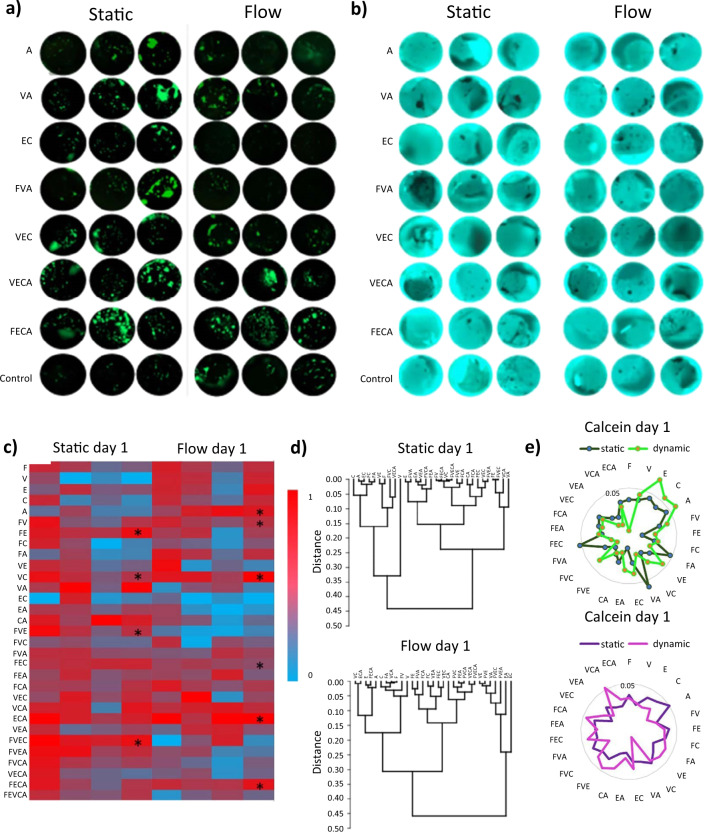
Fluorescence microscopy images of bone marrow-derived MSCs positive for calcein (**a**) alkaline phosphatase (ALP) (**b**) (in black) on biomaterial formulations rendering highest ALP signal ratios and control (protein-free) conditions, after 1 day of cell culture. **c** Heat map representation of ALP present in all scaffolds cultured under static and dynamic conditions, for 1 day of cell culture. **d** Dentograms with hierarchical clustering of the average cell viability values for each formulation and combination of materials. **e** Calculated effects of a 3-factor model for each protein and protein combinations in ALP production and detected calcein under static and dynamic culture conditions. This figure was reproduced with permission from Elsevier (2020)^271^

High-throughput analysis of cell–biomaterials interaction reduces the optimisation time for biomaterial formulation, and allows for an excellent range of material combinations to be trialled. Figure 4 provides an overview of the outputs of a proof-of-concept system of high-throughput analysis that investigated the combination of various ECM-adhesive proteins (fibronectin, collagen I, and vitronectin) under static and flow conditions.^271^ Additionally, computational analysis, including hierarchal clustering and principal component analysis, can aid in optimal formulation discovery by correlating trends in cellular outputs due to the increased sample size being investigated.

One of the largest areas of growth in the biomaterial industry surround composite devices and tissue integration of orthopaedic devices. These devices are typically made from inert metal alloys to sustain the mechanical demands place on them while preventing a foreign body response. Research into material seeks to utilise natural biomaterials, and their native properties to curb foreign body response and improve biological response. Foreign body reaction towards biomaterials occurs upon introduction to bodily fluids and subsequent protein accumulation to the surface of the material.^272^ Integration of the implant with the host tissue is crucial to obtain the desired clinical output. Material coatings often include ECM components of the host tissue to maintain the tissue–device interaction.^272^ Cell attachment onto implant surface, proliferation, and differentiation of these cells improve the formation of new tissue around the device and thereby regeneration.^273^ Collagen type I has been used as a material coating for titanium implants tibial fracture in an in vivo sheep model due to its binding ability to integrin receptors, which enable adherence of osteoblasts and precursor cells.^242,274^ Coating of orthopaedic implants with adhesive molecule fibronectin has also enhanced bone-implant integration via α_5_β_1_ integrin-dependent adhesion and osteogenic differentiation of human mesenchymal stem cells.^275^ Fibronectin and fibronectin-derived oligopeptides have been shown to activate Erk signalling pathway, which involves in the chondrocyte, osteoblast and osteoclast differentiation.^276^ Tropoelastin, the precursor molecule of elastin, interacts with the cells in ECM via elastin-binding receptors such as elastin binding protein, integrin and GAGs.^277^ These interactions enable the tropoelastin molecule to mediate cell differentiation, chemotaxis, and proliferation.^278^ Coating of Ti/Zi metal alloys with tropoelastin has resulted in improved adherence of osteoblasts and osteointegration of the implant.^279^ ECM molecules contain integrin binding motifs, which can be utilised for cell adherence and promote cell–material interaction to induce distinct biological activities through cellular signalling pathways and ensure mechanical stability in the tissue.

Decellularised tissue products have become well established, exemplified by the success of decellularised tissues to create heart valves used in valve replacement surgeries. This technology has seen many successes as well as failures. Bovine and porcine bioprostheses have seen promising results in patients with medium-term follow-up.^280^ On the other hand, translation to cadaveric tissue decellularisation has not been fruitful, marred by variable ECM composition across donors and altered ECM structure and integrity in donors with cardiomyopathy.^281^ Other decellularised tissues used in airway reconstruction have also demonstrated mixed results. While the initial treatment did not require immunosuppression to mitigate host-response to the implant, five-year follow-up data revealed graft stenosis and necessitated intervention.^282^

## Targeted drug delivery through natural biological motifs

Targeted drug delivery is an area of intense research that aims to increase the specificity and efficacy of medication delivery while reducing off-target effects. While an extraordinary range of modalities are currently under investigation, far beyond the scope of this review, we aim to highlight the use of innate biological motifs and potential advantages in drug delivery. In the first section of this review, we highlight the importance of initial material and target tissue characterization to maximize biological activity. Such characterization is also crucial for targeted drug delivery, to incorporate optimal materials for target tissue uptake.

The intrinsic properties of polysaccharides and functional moieties make them ideal delivery vehicles for multiple therapies.^283^ Various forms of HA (nanosized, micellar, or simply HA coating of nanoparticles) have been used as drug delivery system due to its ability to penetrate tissues and tumour sites via enhanced permeability and retention effect, and also its receptor-mediated uptake by cells via CD44 receptor binding,^284^ which is highly expressed in many tumor cells.^285,286^ Besides, HA is highly negatively charged, giving it excellent swelling capacity, and thus allowing a controlled release of drug molecules.^287^ HA has been used in pre-clinical and clinical studies as a carrier for MSC delivery to regenerated the intervertebral disc, augmenting the therapeutic efficacy of MSC therapy due to its anti-inflammatory and anti-apoptotic properties.^288,289^ DS-functionalised nanoparticles are uptaken by melanoma cells via CD146 receptor. This induces caspase-3 cleavage and Poly [ADP-ribose] polymerase 1 pathways are upregulated, promoting tumour apoptosis.^290^ Chondroitin sulphate functionalised nanoparticles have been used to target macrophages expressing CD44 to reduce the inflammation in Ulcerative Colitis, where CS-coated nanoparticles had better biological outcome both in vitro and in vivo versus non-coated counterparts.^291^ Chitosan, on the other hand, is positively charged, an unconventional property for a polysaccharide, allowing its attachment to negatively charged surfaces through electrostatic interactions, such as mucosal membranes. Hence, it can play an essential role in oral, nasal, and ocular drug delivery.^292^

Enzyme-responsive materials are being increasingly studied with increasing complexity. Many of these materials are designed to target tissues with increased enzymatic activity to enhance degradation at the target site. Specific MMP targeting has been used to increase material degradation and drug release in solid tumours with increased expression of MMP-2 and MMP-9.^293,294^ These systems can incorporate multiple targeting modalities including pH-responsive materials, coating shedding, and antigen incorporation to increase targeting even further.^295^

More specific interactions are achievable using materials such as Elastin-b-collagen-like peptide nanovesicles, which target collagen-containing tissues. ELP-CLP nanovesicles show strong adherence on collagen surfaces through collagen triple helix interactions.^296^ Material surfaces can be modified with naturally occurring glycosylation motifs such as mannose disaccharides for subsequent recognition by C-type lectin receptors, predominantly expressed in hepatic non-parenchymal cells.^297^ Furthermore, the brain and lungs can be targeted by adding glucose, increasing the affinity to GLUT1 receptors at the blood-brain barrier and on macrophages.^298^ Integrins are promising candidates for targeted drug delivery using natural peptide motifs. Peptide-drug conjugation has been used to increase integrin α_V_ targeting using an RGD sequence, enhancing doxorubicin delivery to cancer tissues.^299^ This system is limited by the susceptibility of this peptide sequence to hydrolysis of disulphide bonds, eliminating integrin affinity. However, this is a field of intense research with rapidly expanding integrin targets using small molecules and short peptide sequences.

Targeted delivery may be achieved through physical material properties, active receptor motifs, enzyme-responsive materials while efficacy can be increased through simple design considerations. A limitation of targeted delivery using intrinsic properties of materials depends on recognition receptor abundance. For example, while HA has been shown to target specific cancers through CD44 interactions with great success, CD44 is expressed in most cell types making this mechanism inherently non-targeted. While biologically active motifs may aid in the targeted delivery of therapeutics, this will heavily depend on the mode of delivery, potential hepatic metabolism, biodistribution, and target tissue. A combination of targeted delivery techniques from primary sequence manipulation to tertiary structure optimisation may be employed to maximise therapeutic efficacy in addition to other natural and synthetic targeting modalities.

## Future directions/Conclusions

The development of next-generation biomaterials will require a higher understanding of all material properties, both physical and biological, to correctly address the aberrant mechanisms of ECM production, modification, and remodelling in vivo to restore a diseased tissue.^234^ Mammalian-derived polymers possess many biologically active motifs with favourable host-material responses, making them favourable over non-mammalian material sources. It is necessary to assess starting materials and target tissues to consider the biochemical and biomechanical properties, and how they affect/influence cell behaviour and tissue formation, both spatially and temporally to adequately begin to address ECM dysfunction using biomaterial-based therapies. Multiomic approaches to tissue characterisation are making it possible to achieve this level of insight, to develop biomaterial-based implants for complete integration to modulate cell activity and tissue regeneration.^300^ While the cross-linking, assembly and processing techniques suggested in this paper aim to retain the functional biological properties of starting materials, it is impossible to recommend one process over another given the fact that the final application plays a significant role in the necessary material processing. Instead, we highlight the need for further consideration of innate biological activity in material design. Further, assessment of retained bioactivity should be investigated through specific bioassays examining cell–material interaction and intracellular signalling. The selected applications presented here offer insight into a refined approach to material design, through extensive material characterisation and processing, informed by functional assays to assess biological activity.
