# Correction to: Bioactive potential of natural biomaterials: identification, retention and assessment of biological properties

**DOI:** 10.1038/s41392-021-00593-5

**Published:** 2021-05-05

**Authors:** Kieran Joyce, Georgina Targa Fabra, Yagmur Bozkurt, Abhay Pandit

**Affiliations:** 1 School of Medicine, National University of Ireland, Galway, Ireland; 2 CÚRAM, SFI Research Centre for Medical Devices, National University of Ireland, Galway, Ireland

**Keywords:** Biomaterials, Biotechnology

Correction to: *Signal Transduction and Targeted Therapy* 10.1038/s41392-021-00512-8, published online 19 March 2021.

Since the publication of this review article,^1^ the authors recognise a misinterpretation of a cited article in the text. It was previously stated on page 2, column 2, line 54 that: “Silk fibroin binds to receptor activation of nuclear factor κB ligand (RANKL) causing ERK1/2 signalling and expression of NF-κBp65, which promotes induction of osteoclastogenesis.” However, this statement has been corrected to: “Silk fibroin binds to receptor activation of nuclear factor κB ligand (RANKL) inhibiting ERK1/2 signalling and expression of NF-κBp65, and thus inhibits induction of osteoclastogenesis.”

Table 1, Row 6 (“Silk fibroin and sericin”) also incorrectly stated that the silk fibroin based motif for the above mechanism was the GSGAGA sequence, however, no sequence for RANKL binding has yet been definitively identified. This table entry has been updated below to reflect this change. No other findings in this review are affected by these changes.

**Table 1:** Biologically active sequences may be sub-classified into primary sequence recognition sites (in which a receptor directly bind with a specific amino acid sequence or carbohydrate repeats) and secondary and tertiary structures (which depend on molecular conformation, motifs, and domains for recognition).

Macro-molecule	Molecule family	Binding Site/ Functional Motif	Receptor	Activated signalling pathway	Downstream effects	Ref.
Silk Fibroin and Sericin	Insoluble fibrous protein	Unknown	RANKL	Inhibition of ERK1/2 signalling	Osteoclastogenesis inhibition	^2^

Biomaterials contain naturally occurring functional sequences and motifs that bind receptors to induce intracellular signalling and promote downstream effects. (Updated entry only shown here).
